# Supplementary material for: Alcohol-related hepatitis induces a specific fibrosis profile through YAP activation in myofibroblasts
Source: JHEP Rep. 2025 Aug 29;7(12):101580. doi: 10.1016/j.jhepr.2025.101580 (PMC12639322; doi:10.1016/j.jhepr.2025.101580)
Supplement: Multimedia component 1 [file mmc1.pdf]

Alcohol-related hepatitis induces a specific fibrosis profile through YAP activation in myofibroblasts

Line Carolle Ntandja Wandji, Mohamed Bousaleh, Cyril Sobolewski, Mehdi El Amrani, Fabrice Bray, Emmanuel Boleslawski, Jérôme Eeckhoute, Pierre-Jean Devaux, François Maggiotto, Solenne Taront, Christian Rolando, Viviane Gnemmi, Philippe Mathurin, Laurent Dubuquoy, Alexandre Louvet

Table of contents

Supplementary methods……………………………………………………………..2

Supplementary figures…………………………………………………………………4

Supplementary tables…………………………………………………………………..7

Supplementary reference……………………………………………………………..10

Supplementary methods

➢ Isolation of mRNA and real-time PCR

We used a Nucleospin®RNAII kit (Macherey-Nagel, Hoerdt, France) and followed the instructions provided to isolate mRNA from our hepatocytes, myofibroblasts, liver organoids and liver samples. First-strand cDNA was synthesized from 1 µg total RNA, using a High Capacity cDNA Reverse Transcription Kit (Applied Biosystems, Courtaboeuf, France). Real-time PCR was performed using Power SYBR Green PCR Master Mix in a StepOne plus system (both from Applied Biosystems, Foster City, CA, 4 USA). The gene coding for human TATA-binding protein (TBP) was used as housekeeping gene. Specific PCR primers were designed using Primer3 software (https://primer3.org/; **See supplementary Table 1 for details**). The specificity of the amplification and the absence of primer dimerization were ensured by performing a dissociation curve.

➢ Sirius red staining

Deparaffinization and rehydration of the slides were done respectively using the following series of washes: xylene, ethanol and distilled water respectively. Sections were immersed in picro Sirius red solution and stain for 60 min at room temperature. Slides were quickly rinse in acetic acid solution and then dehydrated in absolute alcohol. The slides were mounted under coverslips using resinous mounting medium.

➢ Immunohistochemistry and immunofluorescence

Liver samples fixed with 4% paraformaldehyde were embedded in paraffin. Four-micrometer-thick sections were exposed to primary antibodies overnight (**see Supplementary table 2 for details**) after a specific antigen retrieval step. After washing in Tris buffered saline + 0.05% Tween 20, sections were incubated for 30 min with EnVision+System-HRP Labelled Polymer (Dako Laboratories, Trappes, France) or the EnVision G/2 System/AP Kit for alkaline phosphatase staining. Negative controls were incubated with irrelevant serum or an isotype-matched immunoglobulin instead of the specific antibody. Stained slides were observed under the microscope (Leica, Bensheim, Germany) and analyzed by pathologists.

Organoids cocultured with fibroblasts were embedded in 4% paraformaldehyde without disrupting the BME 2. The same steps above were then realized after permeabilization using Triton 0,01% stored at +4°C.

➢ Proteomic analysis

To identify inflammation/fibrosis-related proteins, significant dysregulated proteins between cirrhosis and AH (adjusted P-value using the Bonferoni-Hochsberg method) were cross-compared with the following human gene sets from the GSEA database (https://www.gsea-msigdb.org/gsea/msigdb/human/genesets.jsp):

HALLMARK_EPITHELIAL_MESENCHYMAL_TRANSITION (M5930), GOBP_FIBROBLAST_MIGRATION (M22676), GOBP_MYOFIBROBLAST_DIFFERENTIATION (M23447), HALLMARK_INFLAMMATORY_RESPONSE (M5932), SA_MMP_CYTOKINE_CONNECTION (M11736), REACTOME_ACTIVATION_OF_MATRIX_METALLOPROTEINASES (M26976), HALLMARK_TGF_BETA_SIGNALING (M5896), GOBP_FIBROBLAST_ACTIVATION (M24593), PLASARI_TGFB1_TARGETS_1HR_UP (M2443), HP_HEPATIC_FIBROSIS (M35270), HP_PERIPORTAL_FIBROSIS (M41235), HP_PORTAL_FIBROSIS (M36886), REACTOME_ACTIVATED_TLR4_SIGNALLING (M15343), GOMF_RAGE_RECEPTOR_BINDING (M18595).

Data were represented as LOG2 fold in a heatmap generated with the Morpheus software (https://software.broadinstitute.org/morpheus/). The mass spectrometry proteomics data have been deposited to the ProteomeXchange Consortium via the PRIDE **(Perez-Riverol Y et *al.* Nucleic Acids Res. 2022)** partner repository with the dataset identifier PXD044031).

➢ Enzyme-linked immunosorbent assay (ELISA)

Cell culture media were collected from wells containing myofibroblasts treated or not treated with GDF 15 (at the concentration of 10 ng/mL). The media underwent centrifugation to remove particulates and assay immediatly. The supernatant was taken to be detected by a

PDGFα/β ELISA kit (Human PDGF-AB, Quantikine ELISA Kit [DHD00C]) by strictly following relevant instructions of kits.

Supplementary figures

- ### Fig. S1:

Comparison of proteomic profiles in perilobular areas from AH vs Cirrh livers

Spatial proteomic analysis shows hierarchical clustering of the most variable proteins between AH and Cirrh extracted from liver perilobular areas

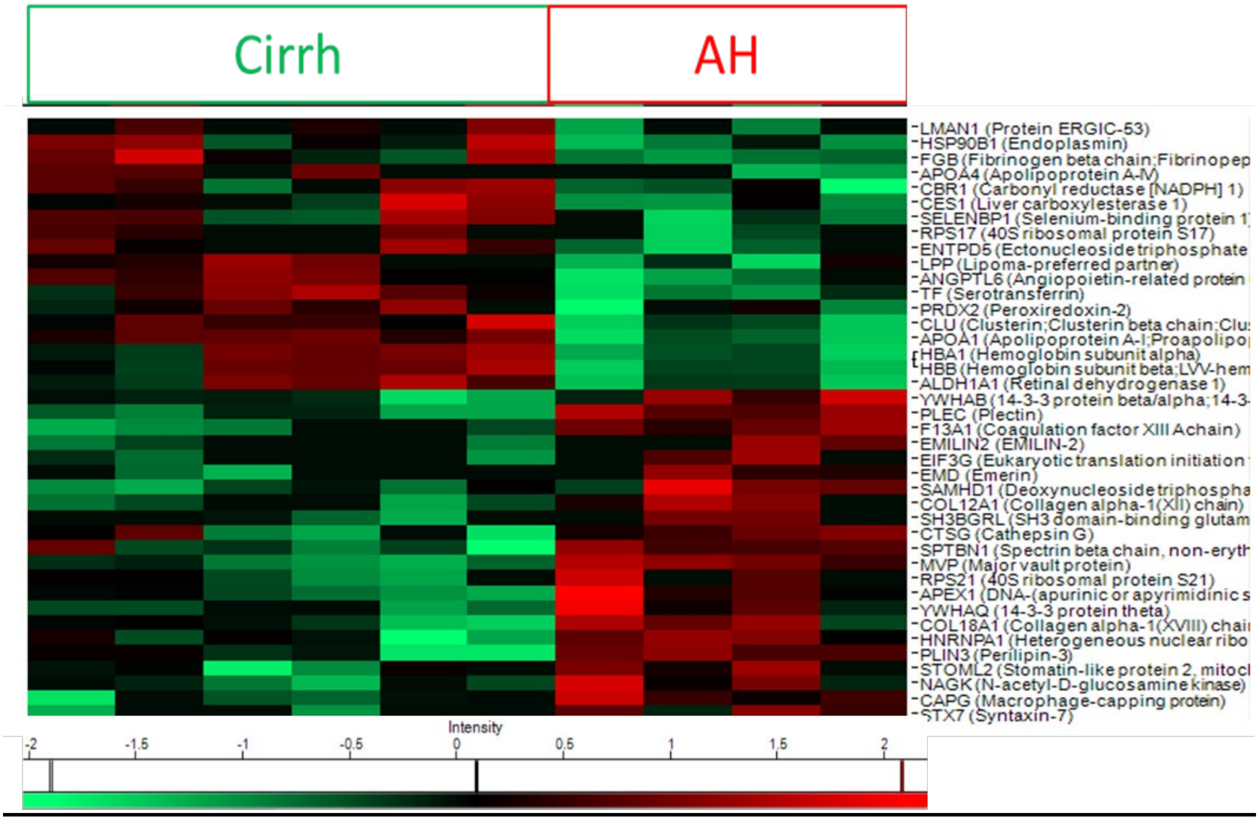

➢ **Fig. S2**

Assessment of CYP3A4 enzymatic activity in organoids obtained from AH or Cirrh livers using the P450-Glo™ assay

The evaluation of CYP3A4 activity in organoids from AH and Cirrh organoids confirms the presence of mature hepatocytes. As expected, CYP3A4 activity was lower in AH organoids than in Cirrh organoids. Statistical significances are indicated (Mann-Whitney U test). * means $p<0.05$; ** means $p<0.01$; *** means $p<0.001$

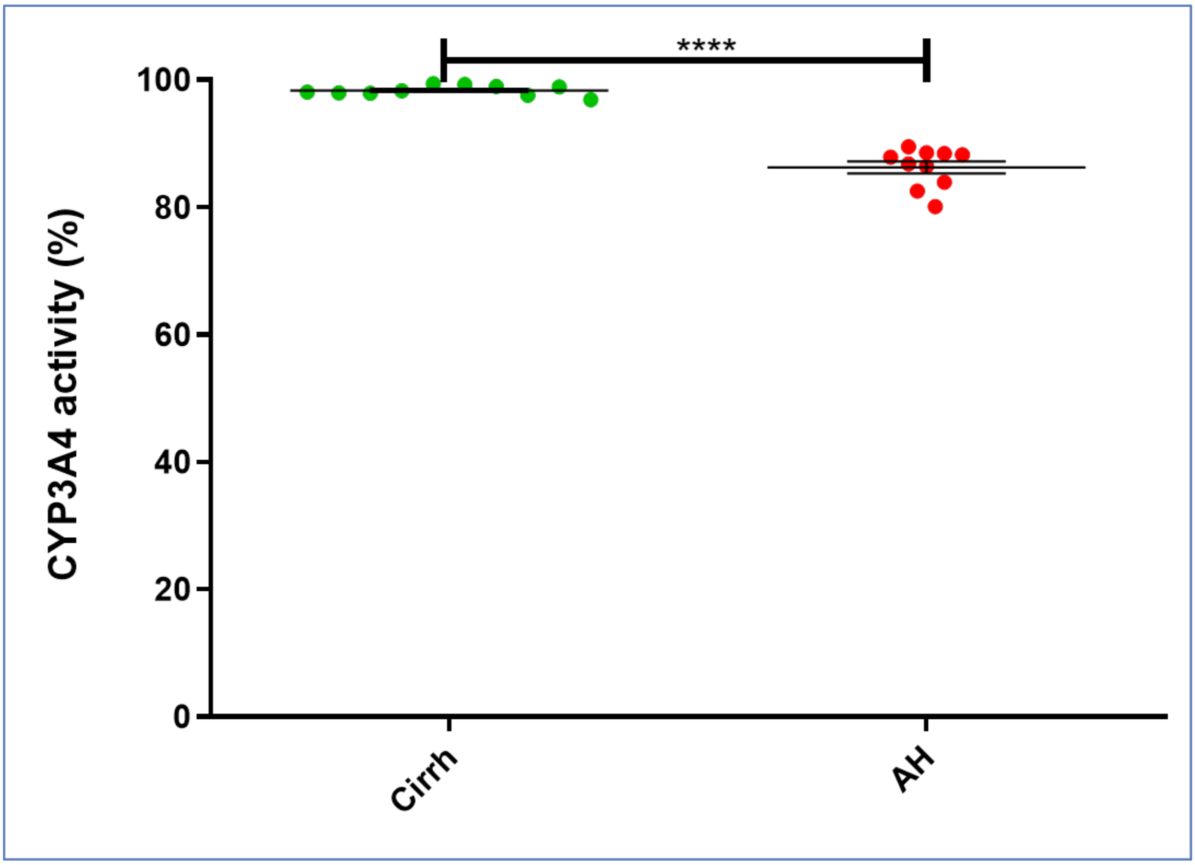

➢ Fig. S3

Expression of YAP and its target genes in transduced and non-transduced Cirrh organoids

Real-time PCRs performed on transduced (with an active YAP, to mimic AH organoids) or non-transduced Cirrh organoids.

mRNA expression levels of the Tag YAPS127A, YAP and its target genes ANKRD1, NUAK2, CYR61 were compared with TBP housekeeping gene (coding for TATA-binding protein)

Statistical significances are indicated (Mann-Whitney U test). * means $p<0.05$; ** means $p<0.01$; *** means $p<0.001$

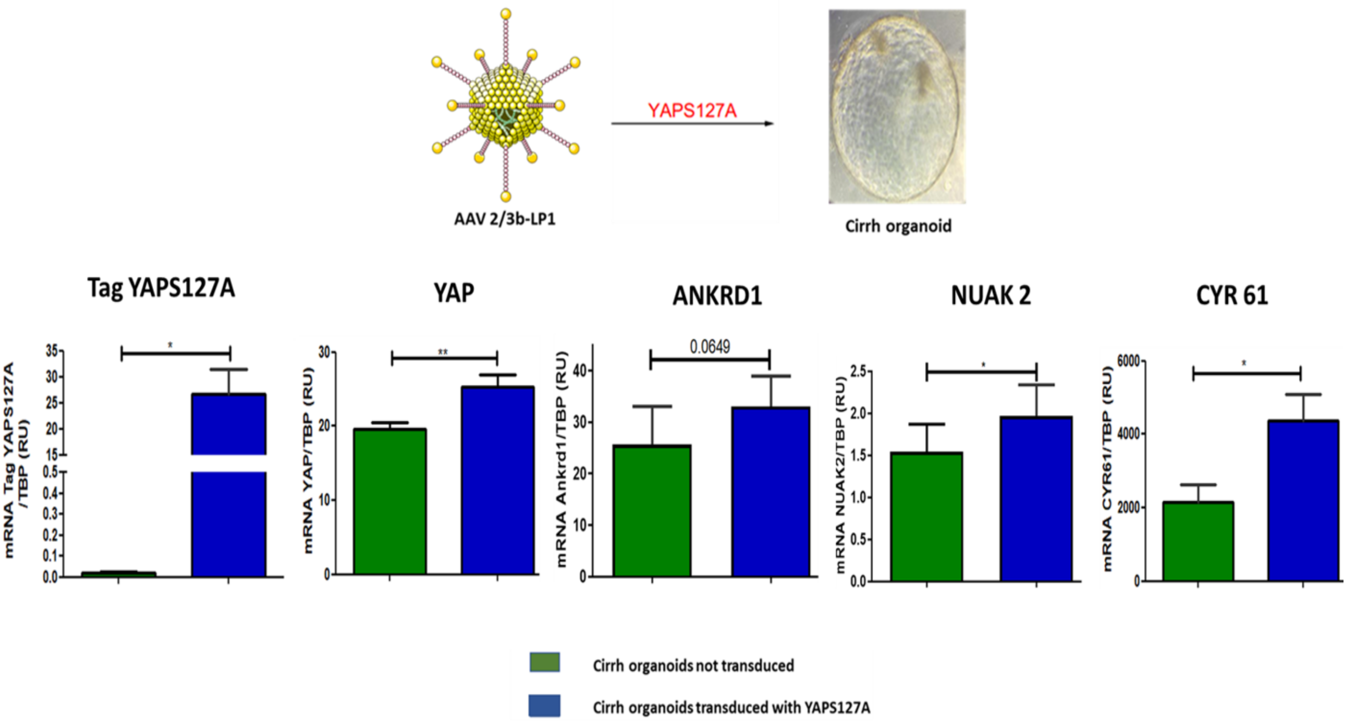

- **Fig. S4**

YAP modulation in human primary hepatocytes

Real-time PCRs performed on transduced (with an active YAP to mimic the aberrant activation of YAP in AH hepatocytes) or non-transduced human primary hepatocytes

mRNA expression levels of YAP, its target genes (ANKRD1, TGFβ2, CYR61, NUAK2 and CTGF), hepatocyte (Albumin, CYP3A4, TAT, Aldolase B) and cholangiocyte (HNF1β, CK 19) markers were compared with TBP housekeeping gene (coding for TATA-binding protein)

Statistical significances are indicated (Mann-Whitney U test). * means $p<0.05$; ** means $p<0.01$; *** means $p<0.001$

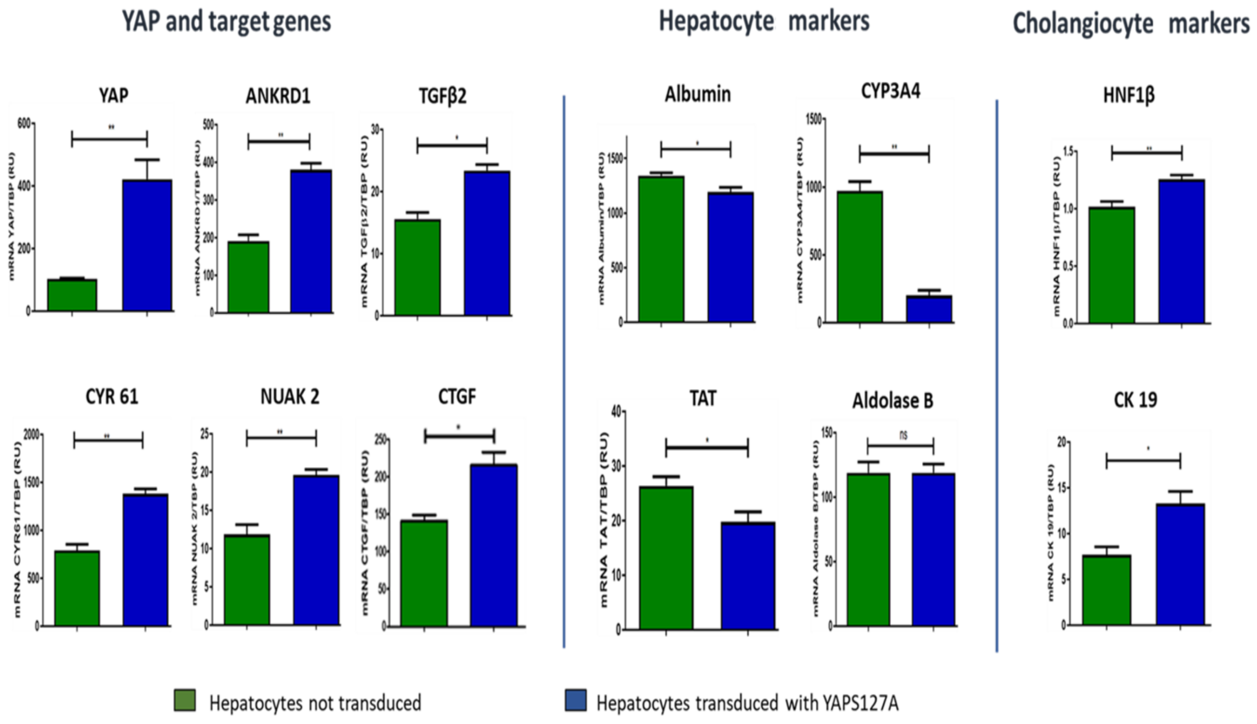

Supplementary tables

- **Table S1**

Primer sequences used for real time RT-PCR

Genes	Forward	Reverse
TBP	TGA-GAA-GAT-GGA-TGT-TGA-GTT-G	AGA-TAG-CAG-CAC-GGT-ATG-AG
αSMA(ACTA2)	CTA-TGC-CTC-TGG-ACG-CAC-AAC-T-	CAG-ATC-CAG-ACG-CAT-GAT-GGC-A
COL1A1	GCC-AAG-ACG-AAG-ACA-TCC-CA	CGT-CAT-CGC-ACA-ACA-CCT-T
PDGFRα	GAT-CTT-TTC-CCT-TGG-TGG-CA	CGT-AGA-CTT-CAC-TGG-TAG-CG
LAMA2	GGC-AAT-CTG-AAT-ACA-CTC-GTG-AC	TGT-GTT-GGT-CCT-CTC-AGC-ATC-C
TIMP1	TGG-AAA-ACT-GCA-GGA-TGG-AC	ATA-AAC-AGG-GAA-ACA-CTG-TGC
MMP9	TTC-TGC-CCG-GAC-CAA-GGA-TA	CCG-GCA-CTG-AGG-AAT-GAT-CT
PDGFα	AGC- GAC-TCC-TGG-AGA-TAG-AC	GAC-AGC-TTC-CTC-GAT-GCT-T
MCP1	GCC-TCC-AGC-ATG-AAA-GTC-TC	AGG-TGA-CTG-GGG-CAT-TGA-T
CCL5	CCTGCTGCTTTGCCTACATTGC	ACACACTTGGCGGTTCTTTCGG
YAP1	TCA-TGC-TTA-GTC-CAC-TGT-CTG-T	TAG-CCC-TGC-GTA-GCC-AGT-TA
Tag YAPS127A	AAGCAAGGCTCGAATCGGTA	TCATGACGGCGTTGAAGAGC
AMOTL2	GCA-AGG-GCT-CTC-TTC-TAG-TG	TGG-GTG-CTC-TGT-CTG-TAG-TC
NUAK2	GAT-GCA-CAT-ACG-GAG-GGA-GATT	ATC-ACG-ATC-TTG-CTG-CTG-TTC-T
TGFβ2	CAC-GAA-CCC-AAA-GGG-TAC-AA	ATA-TAA-GCT-CAG-GAC-CCT-GCT
CTGF	TGG-AGG-AAA-ACA-TTA-AGA-AGG-G	AAG-CTC-AAA-CTT-GAC-AGG-CT
CYR61	TTG-GTA-ACT-CGT-GTG-GAG-ATG	GAA-GAG-GCT-TCC-TGT-CTT-TGG
CYCLIN D1	TGC-ATG-TTC-GTG-GCC-TCT-AAG	TCG-GTG-TAG-ATG-CAC-AGC-TTC-T

- **Table S2**

Antibodies used in immunostaining

Name	Host	Supplier	Reference
**αSMA**	Mouse	DAKO	M0851
**COL1A1**	Mouse	Sigma-Aldrich	SAB1402151
**PDGFRα**	Rabbit	Cell signaling	5241
**YAP**	Rabbit	Cell signaling	≠ 14074
**Albumin**	Goat	Bethyl laboratory	A80-229A
**GDF 15**	Rabbit	Cell signaling	≠ 79996

Supplementary reference

1- Perez-Riverol Y, Bai J, Bandla C, et al. The PRIDE database resources in 2022: a hub for mass spectrometry-based proteomics evidences. *Nucleic Acids Res*. 2022;50(D1):D543-D552. doi:10.1093/nar/gkab1038
