# Supplementary material for: Alcohol-related hepatitis induces a specific fibrosis profile through YAP activation in myofibroblasts
Source: JHEP Rep. 2025 Aug 29;7(12):101580. doi: 10.1016/j.jhepr.2025.101580 (PMC12639322; doi:10.1016/j.jhepr.2025.101580)
Supplement: Multimedia component 2 [file mmc2.docx]

**JHEP Reports**

**CTAT methods**

Tables for a “Complete, Transparent, Accurate and Timely account” (CTAT) are now mandatory for all revised submissions. The aim is to enhance the reproducibility of methods.

- Only include the parts relevant to your study
- Refer to the CTAT in the main text as ‘Supplementary CTAT Table’
- Do not add subheadings
- Add as many rows as needed to include all information
- Only include one item per row

**If the CTAT form is not relevant to your study, please outline the reasons why:**

|  |
| --- |

- 1. **Antibodies**

| **Name** | **Citation** | **Supplier** | **Cat no.** | **Clone no.** |
| --- | --- | --- | --- | --- |
| **αSMA** |  | DAKO | M0851 |  |
| **COL1A1** |  | Sigma-Aldrich | SAB1402151 |  |
| **PDGFRα** |  | Cell signaling | 5241 |  |
| **YAP** |  | Cell signaling | #14074 | D8H1X |
| **Albumin** |  | Bethyl laboratory | A80-229A |  |
| **GDF 15** |  | Cell signaling | #79996 |  |

- 1. **Cell lines**

| **Name** | **Citation** | **Supplier** | **Cat no.** | **Passage no.** | **Authentication test method** |
| --- | --- | --- | --- | --- | --- |
|  |  |  |  |  |  |

- 1. **Organisms**

| **Name** | **Citation** | **Supplier** | **Strain** | **Sex** | **Age** | **Overall n number** |
| --- | --- | --- | --- | --- | --- | --- |
|  |  |  |  |  |  |  |

- 1. **Sequence based reagents**

| **Name** | **Sequence** | **Supplier** |
| --- | --- | --- |
| **TBP for** | **TGA-GAA-GAT-GGA-TGT-TGA-GTT-G** | Eurogentec |
| **TBP rev** | **AGA-TAG-CAG-CAC-GGT-ATG-AG** | Eurogentec |
| **ACTA2 for** | **CTA-TGC-CTC-TGG-ACG-CAC-AAC-T** | Eurogentec |
| **ACTA2 rev** | **CAG-ATC-CAG-ACG-CAT-GAT-GGC-A** | Eurogentec |
| **COL1A1 for** | **GCC-AAG-ACG-AAG-ACA-TCC-CA** | Eurogentec |
| **COL1A1 rev** | **CGT-CAT-CGC-ACA-ACA-CCT-T** | Eurogentec |
| **PDGFRA for** | **GAT-CTT-TTC-CCT-TGG-TGG-CA** | Eurogentec |
| **PDGFRA rev** | **CGT-AGA-CTT-CAC-TGG-TAG-CG** | Eurogentec |
| **LAMA2 for** | **GGC-AAT-CTG-AAT-ACA-CTC-GTG-AC** | Eurogentec |
| **LAMA2 rev** | **TGT-GTT-GGT-CCT-CTC-AGC-ATC-C** | Eurogentec |
| **TIMP1 for** | **TGG-AAA-ACT-GCA-GGA-TGG-AC** | Eurogentec |
| **TIMP1 rev** | **ATA-AAC-AGG-GAA-ACA-CTG-TGC** | Eurogentec |
| **MMP9 for** | **TTC-TGC-CCG-GAC-CAA-GGA-TA** | Eurogentec |
| **MMP9 rev** | **CCG-GCA-CTG-AGG-AAT-GAT-CT** | Eurogentec |
| **PDGFA for** | **AGC- GAC-TCC-TGG-AGA-TAG-AC** | Eurogentec |
| **PDGFA rev** | **GAC-AGC-TTC-CTC-GAT-GCT-T** | Eurogentec |
| **MCP1 for** | **GCC-TCC-AGC-ATG-AAA-GTC-TC** | Eurogentec |
| **MCP1 rev** | **AGG-TGA-CTG-GGG-CAT-TGA-T** | Eurogentec |
| **CCL5 for** | **CCTGCTGCTTTGCCTACATTGC** | Eurogentec |
| **CCL5 rev** | **ACACACTTGGCGGTTCTTTCGG** | Eurogentec |
| **YAP1 for** | **TCA-TGC-TTA-GTC-CAC-TGT-CTG-T** | Eurogentec |
| **YAP1 rev** | **TAG-CCC-TGC-GTA-GCC-AGT-TA** | Eurogentec |
| **Tag YAPS127A for** | **AAGCAAGGCTCGAATCGGTA** | Eurogentec |
| **Tag YAPS127A rev** | **TCATGACGGCGTTGAAGAGC** | Eurogentec |
| **AMOTL2 for** | **GCA-AGG-GCT-CTC-TTC-TAG-TG** | Eurogentec |
| **AMOTL2 rev** | **TGG-GTG-CTC-TGT-CTG-TAG-TC** | Eurogentec |
| **NUAK2 for** | **GAT-GCA-CAT-ACG-GAG-GGA-GATT** | Eurogentec |
| **NUAK2 rev** | **ATC-ACG-ATC-TTG-CTG-CTG-TTC-T** | Eurogentec |
| **TGFB2 for** | **CAC-GAA-CCC-AAA-GGG-TAC-AA** | Eurogentec |
| **TGFB2 rev** | **ATA-TAA-GCT-CAG-GAC-CCT-GCT** | Eurogentec |
| **CCN2 for** | **TGG-AGG-AAA-ACA-TTA-AGA-AGG-G** | Eurogentec |
| **CCN2 rev** | **AAG-CTC-AAA-CTT-GAC-AGG-CT** | Eurogentec |
| **CCN1 for** | **TTG-GTA-ACT-CGT-GTG-GAG-ATG** | Eurogentec |
| **CCN1 rev** | **GAA-GAG-GCT-TCC-TGT-CTT-TGG** | Eurogentec |
| **CCND1 for** | **TGC-ATG-TTC-GTG-GCC-TCT-AAG** | Eurogentec |
| **CCND1 rev** | **TCG-GTG-TAG-ATG-CAC-AGC-TTC-T** | Eurogentec |
| **KRT19 for** | **TTT-GAG-ACG-GAA-CAG-GCT-CT** | Eurogentec |
| **KRT19 rev** | **CCC-TCA-GCG-TAC-TGA-TTT-CC** | Eurogentec |
| **HNF1B for** | **GAT-CAC-AGT-GTC-GGG-AGG-A** | Eurogentec |
| **HNF1b rev** | **GTG-TTG-AGG-CTC-TGT-GCA-AT** | Eurogentec |

- 1. **Biological samples**

| **Description** | **Source** | **Identifier** |
| --- | --- | --- |
| **Human liver sample** | **Biobank** | **TargetOH** |
| **Primary isolated hepatocytes** | **Biopredic, Rennes, France** |  |

- 1. **Deposited data**

| **Name of repository** | **Identifier** | **Link** |
| --- | --- | --- |
|  |  |  |

- 1. **Software**

| **Software name** | **Manufacturer** | **Version** |
| --- | --- | --- |
| **Graphpad Prism** | **GraphPad Software Inc** | **5.03** |
| **RStudio** |  | **4.2.1** |
| **Inkscape** | **General Public License** | **1.0.1-** |
| **Leica Application Suite** | **Leica Microsystems** | **LAS V3.7** |
| **Image J software** | **NIH** | **1.52h** |
| **Office 2010** | **Microsoft** | **14.0.7015.1000** |
| **StepOne software** | **Applied Biosystems** | **2.3** |

- 1. **Other (*e.g*. drugs, proteins, vectors etc.)**

| **Name** | **Supplier** | **Cat no.** |
| --- | --- | --- |
| **carboxyfluorescein diacetate succinimidyl ester (CFSE)** | **Invitrogen, Carlsbad, CA, USA** | **C34570** |
| **AAV 2/3b-LP1- YAPS127A** | **UMR1089 – CPV lab** | **N/A** |
| **P450-Glo™ CYP3A4 Assay** | **Promega, France** | **V8911** |
| **Basement Membrane Extract Type 2** | **Amsbio, UK** | **3533-005-02** |
| **CryoStor®** | **Sigma-Aldrich** | **C2999** |
| **EPCAM sorting** | **Milteny Biotec** | **130-061-101** |

- 1. **Please provide the details of the corresponding methods author for the manuscript:**

| **Line Carolle NTANDJA WANDJI, INFINITE-U1286, Faculté de Médecine – Pole Recherche, 1 Place Verdun, 59045 Lille Cedex, France**  **Line.NTANDJAWANDJI@chu-lille.fr** |
| --- |

**2.0 Please confirm for randomised controlled trials all versions of the clinical protocol are included in the submission. These will be published online as supplementary information.**

| **N/A** |
| --- |
