# Supplementary material for: Alcohol-related hepatitis induces a specific fibrosis profile through YAP activation in myofibroblasts
Source: JHEP Rep. 2025 Aug 29;7(12):101580. doi: 10.1016/j.jhepr.2025.101580 (PMC12639322; doi:10.1016/j.jhepr.2025.101580)
Supplement: Multimedia component 4 [file mmc4.pdf]

Research article

JHEP|Reports

Alcohol-related hepatitis induces a specific fibrosis profile through YAP activation in myofibroblasts

Authors

Line Carolle Ntandja Wandji, Mohamed Bou Saleh, Cyril Sobolewski, …, Viviane Gnemmi, Philippe Mathurin, Laurent Dubuquoy†, Alexandre Louvet†

Correspondence

laurent.dubuquoy@inserm.fr (L. Dubuquoy), alexandre.louvet@chru-lille.fr (A. Louvet).

Graphical abstract

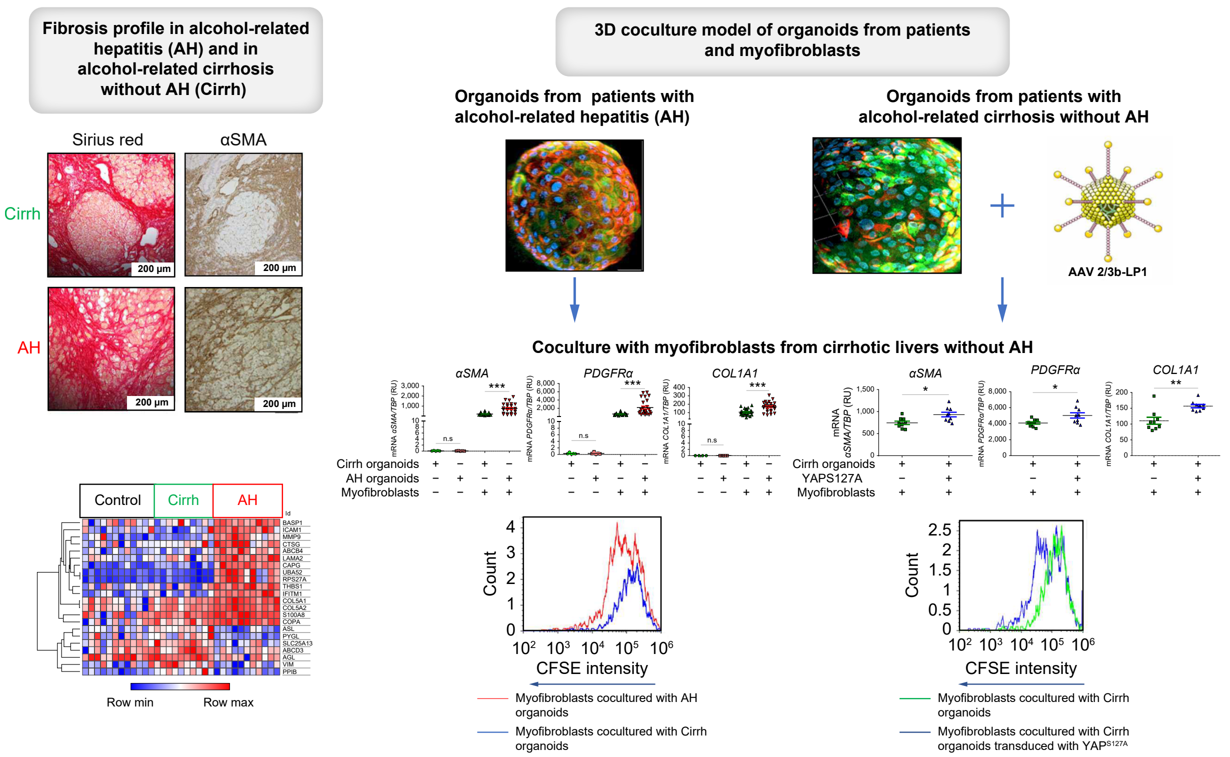

Highlights:

- AH is associated with a specific fibrosis profile and protein signature.
- Aberrant YAP activation in altered hepatocytes is involved in the activation and proliferation of myofibroblasts in AH livers.
- In the 2D model, YAP-conditioned medium induced increased myofibroblast activation and proliferation, in part, through GDF15.
- In a 3D co-culture model, AH organoids increased activation and proliferation of myofibroblasts compared with Cirrh organoids.

Impact and implications:

Aberrant activation of YAP in hepatocytes contributes to impaired regeneration in alcohol-related hepatitis (AH) by driving their transdifferentiation toward a cholangiocyte-like phenotype. Preliminary data suggest that the extracellular matrix in AH differs from cirrhosis, with an increased laminin-to-fibronectin ratio. In a 3D co-culture model of liver organoids and myofibroblasts, AH organoids increased activation and proliferation of myofibroblasts compared with cirrhosis organoids. Aberrant YAP activation in altered hepatocytes is involved in the activation and proliferation of myofibroblasts in AH livers. Thus, inhibiting YAP in hepatocytes could be an attractive approach for the development of AH treatments to improve hepatic regeneration and decrease fibrogenesis.

https://doi.org/10.1016/j.jhepr.2025.101580
© 2025 The Author(s). Published by Elsevier B.V. on behalf of European Association for the Study of the Liver (EASL). This is an open access article under the CC BY license (http://creativecommons.org/licenses/by/4.0/). JHEP Reports, 2025, 7, 1–12

Research article

JHEP Reports

Alcohol-related hepatitis induces a specific fibrosis profile through YAP activation in myofibroblasts

Line Carolle Ntandja Wandji[1,2], Mohamed Bou Saleh[1], Cyril Sobolewski[1], Mehdi El Amrani[3,4], Fabrice Bray[5], Emmanuel Boleslawski[4,6], Jérôme Eeckhoute[7], Pierre-Jean Devaux[1], François Maggiotto[1], Solenne Taront[1], Christian Rolando[5], Viviane Gnemmi[3,8], Philippe Mathurin[1,2], Laurent Dubuquoy[1,*,†], Alexandre Louvet[1,2,*,†]

JHEP Reports **2025**. vol. 7 | 1–12

Check for updates

Background & Aims: Yes-associated protein (YAP) impairs hepatocyte regeneration in alcohol-related hepatitis (AH), but its impact on fibrogenesis remains to be characterized. Our aim here was to describe fibrogenesis and investigate the impact of altered hepatocytes on fibrogenic mechanisms during AH.

Methods: Fibrosis, YAP and alpha smooth muscle actin (αSMA) distribution were assessed by immunostaining. Extracellular matrix (ECM) composition in severe AH (n = 22), alcohol-related cirrhosis without AH (Cirrh, n = 24), and healthy livers (CTRL, n = 15) was assessed by PCR, and bulk and spatial proteomics. The impact of organoids generated from AH and Cirrh livers on myofibroblasts was also evaluated. Cirrh organoids were transduced with constitutively active YAP (to mimic AH organoids) and co-cultured with myofibroblasts in a 3D co-culture model. In a 2D model, myofibroblasts were treated with growth differentiation factor 15 (GDF15) or cultured with medium from transduced (YAP medium) and non-transduced hepatocytes.

Results: AH livers presented with both perilobular (as in Cirrh livers) and specifically intralobular fibrosis. Bulk proteomics and PCR showed a specific ECM protein signature (*e.g.* laminin A2 was increased (p <0.05) whereas vitronectin was decreased (p <0.001)) in AH. Spatial proteomics showed differences in ECM composition between intralobular and perilobular areas in AH and Cirrh livers. AH organoids also overexpressed YAP (p <0.01). In the 3D model, AH organoids induced greater activation (*e.g. αSMA* mRNA level increased by threefold; p <0.001) of co-cultured myofibroblasts. YAP-transduced Cirrh organoids also induced greater activation of co-cultured myofibroblasts: for example, the *αSMA* mRNA level increased by 1.5-fold (p <0.05). In the 2D model, YAP medium also induced increased myofibroblast activation (*αSMA* mRNA level increased by 1.5-fold; p <0.01) and proliferation (23% increase; p <0.05), in part, through GDF15.

Conclusion: AH displays a specific fibrosis profile and protein signature. AH organoids and YAP-transduced Cirrh organoids induce myofibroblast activation. Thus, hepatocyte YAP activation has an important role in the fibrogenesis observed in AH.

© 2025 The Author(s). Published by Elsevier B.V. on behalf of European Association for the Study of the Liver (EASL). This is an open access article under the CC BY license (http://creativecommons.org/licenses/by/4.0/).

Introduction

Alcohol-related liver disease (ARLD) remains one of the leading causes of chronic liver disease worldwide. The therapeutic options of alcohol-related hepatitis (AH), the most severe form of ARLD, are limited because our understanding of its pathophysiology has been slowed by a lack of reliable animal models and insufficient access to liver samples of patients with AH. Early liver transplantation is the only treatment associated with good short- and long-term survival in highly selected patients[1–4] who do not respond to steroids, the standard treatment in patients with severe AH.[5] However, because of organ shortages, new therapeutic options are urgently needed and new pathways and targets must be identified.[6]

Emerging therapies under evaluation for AH involve targeting inflammation,[7,8] the gut–liver axis, apoptotic pathways, and liver regeneration, but have achieved disappointing results so far. Given that the degree of fibrosis is a key driver of outcome in patients with AH and alcohol-related cirrhosis without AH (Cirrh),[9,10] treatment strategies focusing on the fibrogenic process are also attractive. Although damage-associated molecular patterns and oxidative stress, which activate Kupffer cells, have a role in the activation of hepatic stellate cells (HSCs),[10–12] the mechanisms underlying the activation of HSCs in AH have not been extensively studied. Preliminary data suggest that the composition of the extracellular matrix (ECM) is modified in AH livers,[13] with an increased laminin/fibronectin ratio associated with laminin surrounding hepatic progenitor cells.[13] Therefore, we hypothesize that there is a possible direct interaction between these two cell types. Furthermore, although human data are lacking, data from murine models suggest that a modified ECM influences the fate of progenitor cells by decreasing their differentiation in hepatocytes.[14,15]

* Corresponding author. Address: Service des Maladies de l'Appareil Digestif, Hôpital Huriez, Rue Michel Polonovski, F-59037 Lille cedex France. Tel.: + 33 3 20 44 53 03; Fax 33 3 20 44 55 64 AND Univeristy of Lille, Inserm, CHU Lille, U1286 - INFINITE - Institute for Translational Research in Inflammation, F-59000 Lille, France
E-mail addresses: laurent.dubuquoy@inserm.fr (L. Dubuquoy), alexandre.louvet@chru-lille.fr (A. Louvet).
† Co-last authors.
https://doi.org/10.1016/j.jhepr.2025.101580

Aberrant YAP activation in AH hepatocytes leads to their transdifferentiation toward a cholangiocyte-like phenotype and, thus, impaired regeneration.[16] Data from murine models have also shown that YAP activates myofibroblasts and, thus, could interfere with fibrogenesis.[17,18] We investigated the specific effect of aberrant YAP activation in hepatocytes on myofibroblast activation, resulting in a specific profile of fibrogenesis in AH.

With their ability to conserve the phenotypic characteristics of *in vivo* tissues, organoids are innovative tools that can help understand the mechanisms involved in chronic liver diseases.[19,20] To our knowledge, there are no organoids available from patients with AH. However, AH organoids could be used to improve our understanding of the underlying mechanisms of AH.

The goals of this study were to characterize the fibrosis profile of AH (compared with Cirrh) to confirm the specific fibrosis profile of this disease and to investigate the relationship, independently of the inflammatory component, between aberrant YAP activation in hepatocytes and myofibroblast activation resulting in the specific fibrogenesis profile of AH.

Patients and methods

Patients

This study was authorized by the ethics committee of Hôpital Huriez, Lille, France. Clear and appropriate information was provided to all patients, who gave their consent before data collection (2014-A01452-45, NCT03773887). Liver samples were collected from patients who underwent early liver transplantation for severe AH (defined by a Maddrey discriminant function >32) (AH, n = 22) that did not respond to steroids (defined by a Lille model score >0.45) at the Hepatology Unit of Hôpital Huriez. Before steroids were initiated, a biopsy-proven diagnosis of AH was made using routine French protocols. After liver transplantation, all liver samples from explants were histologically analyzed by an expert pathologist (VG) and fibrosis was classified by the SALVE score[9] (which is considered the gold standard for grading fibrosis in patients with alcohol-related liver disease). Patients with Cirrh (n = 24) who underwent transplants for decompensated cirrhosis were used as disease controls. Healthy liver samples were collected from patients who underwent hepatic resection for colorectal cancer metastases (CTRL, n = 15). Liver samples were immediately fixed or frozen for all experiments except cell isolation.

The clinical and biological characteristics of included patients are described in Table 1. As expected, patients with severe AH who did not respond to steroids had more severe liver dysfunction, including higher bilirubin levels (237 *vs.* 67 mg/L, p <0.0001), model for end-stage liver disease (MELD) scores (26 *vs.* 21.5, p = 0.002), serum creatinine (p = 0.02), and C reactive protein (CRP) levels (29 *vs.* 10 mg/L, p = 0.005) compared with patients with Cirrh. All patients (AH and Cirrh) had histological cirrhosis.[13,21] Half of the patients (11/22) with AH had incomplete cirrhosis (stage 4A) and the other half had cirrhosis with broad (4B) or very broad septa (4C). Only 10% of patients with Cirrh had incomplete cirrhosis (4A), whereas 90% of patients with Cirrh had cirrhosis with broad or very broad septa. When considering pericellular fibrosis (PCF), 90% of patients with AH had PCF, compared with only 10% of those with Cirrh.

Table 1. Clinical characteristics of patients in the three study groups.

Characteristic	CTRL patients (n = 15)	Patients with Cirrh (n = 24)	Patients with AH (n = 22)	*p* value*
INR	N/A	1.75	1.9	0.494
Serum creatinine, (mg/dl)	0.7	0.8	1.6	**0.021**
Serum albumin (g/L)	42	30	26	0.2
Bilirubin (mg/dl)	0.3	6.7	23.7	**<0.0001**
CRP (mg/L)	9	10	29	**0.005**
MELD score	N/A	21.5	26	**0.002**

Data are given as medians; *comparison between patients with Cirrh and those with AH; values in bold denote statistical significance. AH, alcohol-related hepatitis; Cirrh, alcohol-related cirrhosis without AH; CRP, C-reactive protein; CTRL, control; INR, international normalized ratio; MELD, model for end-stage liver disease.

Isolation and culture of primary human hepatocytes and myofibroblasts

As previously described by our group,[16] human hepatocytes were isolated from CTRL liver resection using a specific perfusion kit containing, among other compounds, EDTA/collagenase (Biopredic, Rennes, France). Briefly, the parenchymal fraction was isolated after perfusion of the liver using this kit. The cell suspension obtained after this perfusion was filtered through different membranes (100 µm, 70 µm, and 40 µm) and centrifuged three times at 210 *g* for 2 min at 4 °C. The pellet containing the hepatocytes was harvested in the seeding medium (Biopredic). Cell viability was evaluated in a Thoma counting chamber, using Trypan Blue staining. The hepatocytes were then cultured in collagen-coated six-well plates (354400, Dutscher, Brumath, France) at 37 °C in humidified 5% CO_2.

The myofibroblast-containing supernatant obtained after previous centrifugations was centrifugated a further three times at 800 *g* for 5 min at 8 °C. The pellet containing myofibroblasts was cultured in a cell culture flask T-175 (2582569, Sarstedt, France) with DMEM 1x high glucose + GlutaMAX™ ((Life Technologies, cat. no. 31966-021, Grand Island, New York, USA) supplemented with human serum type AB (10%) and 1% streptomycin and penicillin at 37 °C in humidified 5% CO_2. After 72 h, these cultured cells were trypsinized and fluorescence-activated cell sorting (FACS) analysis confirmed the presence of >90% of myofibroblasts marked by THY1 with a viability >95%. Myofibroblasts isolated from CTRL (n = 2) and Cirrh livers (n = 3) were used in this study.

Human organoids obtained from AH or Cirrh livers

Samples from explanted livers (AH, n = 5; Cirrh, n = 5) were kept frozen at -80 °C in a cryovial tube containing 500 µl of CryoStor® (C2999 Sigma-Aldrich, Saint Quentin Fallavier, France) until use. After defrosting, cells were dissociated using collagenase D (2.5 mg/ml, Sigma-Aldrich, C9407, Darmstadt, Germany) at 37 °C for 5–10 min according to the sample size. After filtration (100 µm and 70 µm) and centrifugation (200 ***g*** for 5 min at 4 °C), the progenitor cells were then isolated by EpCAM sorting (Milteny Biotec, 130-061-101, Paris, France). Sorted cells were embedded in an artificial extracellular matrix (Basement Membrane Extract (BME) Type 2 (3533-005-02

Amsbio, Francfort, Germany)) and organoids were differentiated using cocktails of cytokines based on the protocol described by Broutier *et al.*[22]

Assessment of CYP3A4 enzymatic activity in human organoids obtained from AH or Cirrh livers using the P450-Glo™ assay

Organoids generated from 1,000 EpCAM-positive epithelial cells isolated from AH and Cirrh livers were seeded into white 96-well plates suitable for luminescence detection. Organoids were cultured for 5 days in expansion medium, followed by 14 days in differentiation medium to induce functional maturation. CYP3A4 enzymatic activity was assessed using the P450-Glo™ CYP3A4 Assay with Luciferin-PPXE substrate (Promega, V8911, Madison, WI, USA) according to the manufacturer's protocol. After the differentiation period, organoids were incubated with the Luciferin-PPXE substrate to allow CYP3A4-dependent conversion. Subsequently, a detection reagent was added, and luminescence was measured using a microplate luminometer. Relative CYP3A4 activity was quantified and compared between AH- and Cirrh-derived organoids to assess differences in xenobiotic metabolism capacity.

Development of a 3D co-culture model of organoids and myofibroblasts

To study the direct impact of altered hepatocytes on myofibroblasts, we developed an *ex vivo* 3D co-culture model of organoids (containing exclusively epithelial cells) and myofibroblasts. Given that we wanted to explore a fibrogenesis mechanism that was independent of the inflammatory component, we excluded from our model the main cells of the inflammatory process, such as Kupffer cells and neutrophils. Using the protocol described by Broutier *et al.*,[22] organoids were cultured in human liver expansion medium until they were 80% confluent. After disrupting the basement matrix containing the organoids, the latter were trypsinized to single cells. After verifying the viability of these single cells, human primary hepatic myofibroblasts (which had undergone six passages at most) were added to obtain a ratio of nine myofibroblasts per single cell. BME2 was added to the cellular mixture. The set was then cultured for 5 days in the human expansion medium and 10 days in the human differentiation medium. RNA was then isolated to evaluate the myofibroblast activation.

YAP modulation in human hepatocytes and organoids and evaluation of its impact on myofibroblast activation

To evaluate the impact of aberrant YAP activation in altered hepatocytes from patients with AH on the activation of myofibroblasts, freshly isolated human hepatocytes from healthy livers were plated at 3.8×10^5/well in a collagen-coated 24-well plate and then cultured in William's E medium supplemented with 100 U/ml penicillin, 100 μg/ml streptomycin, 4 μg/ml bovine insulin and 50 μM hydrocortisone hemisuccinate. Transduction of these hepatocytes (to mimic the aberrant activation of YAP observed in AH altered hepatocytes) was achieved using a vector to enable strong expression of a constitutively active human YAP protein mutant (*YAP*^{S127A}), which cannot be inhibited by Hippo signaling. Details of the preparation and purification of the vector (AAV 2/3b-LP1-*YAP*^{S127A}) have been published elsewhere.[16] The medium was changed 3 days after the start of transduction. Primary hepatocytes were recovered at 6 days and the conditioned medium was collected from the wells.

Myofibroblasts isolated from CTRL livers (n = 2) were also cultured. After obtaining 80% confluence, human primary myofibroblasts were starved for 24 h, then cultured with conditioned medium collected from wells containing transduced (YAP medium) or non-transduced hepatocytes (CTRL medium). The paracrine effect of YAP modulation in hepatocytes on the activation of myofibroblasts was then evaluated.

The vector AAV 2/3b-LP1-*YAP*^{S127A} was also used to transduce organoids from patients with Cirrh (Cirrh organoids) to mimic organoids obtained from patients with AH (AH organoids). We used 10^5 Vg/cells of adeno-associated virus (AAV) in the expansion medium, which was not changed for 3 days. We then differentiated the organoids. YAP-transduced or non-transduced Cirrh organoids were cultured with primary human liver myofibroblasts to evaluate their activation through direct interaction and, thus, also the impact of hepatocyte YAP.

Evaluation of myofibroblast proliferation in a 3D co-culture model with organoids

Myofibroblast cells used in the 3D co-culture model were first labeled with carboxyfluorescein diacetate succinimidyl ester (CFSE) (Invitrogen, Carlsbad, CA, USA) to evaluate their proliferation. Briefly, myofibroblasts were starved for 6 h and then incubated at 37 °C for 20 min with prewarmed PBS containing 10 μM CFSE. After incubation, myofibroblasts were washed with medium three times and co-cultured with organoids. After 14 days of co-culture, the cells were trypsinized and collected. Dead cells were isolated using 7-amino-actinomycin D. Myofibroblasts in viable cells were isolated using THY1 antibodies and analyzed by flow cytometry using Kaluza software (v2.3, Beckman Coulter, Brea, California, USA). Lower CFSE intensity reflected greater myofibroblast proliferation.

Proteomic analysis

Proteins were extracted with RIPA buffer after homogenization of liver samples (11 CTRL, nine Cirrh, and 12 AH) with a tissue homogenizer for proteomic analysis. Soluble proteins were recovered from the supernatants and insoluble proteins were extracted from the pellets after another RIPA extraction. The proteins were then digested with trypsin by the eFASP method.[23] The resulting peptides were analyzed by liquid chromatography (LC)-mass spectrometry (MS)/MS on a Q Exactive plus mass spectrometer.[24] The proteins were quantified and identified using MaxQuant software (v 2.0.3.0) with the reviewed *Homo sapiens* UniProt database (release May 2022). Statistical analysis of the identified proteins was performed with Perseus (v 2.0.3.0),[25] the Student's *t* test, and ANOVA; $p < 0.05$ was considered to be statistically significant.

Proteins from conditioned media collected from wells containing transduced and non-transduced hepatocytes or myofibroblasts treated with GDF15 at a concentration of 10 ng/ml (Gibco™ Human GDF-15/MIC-1 Recombinant Protein, PeproTech, Neuilly-Sur-Seine, France), were also extracted and quantified, using the same method.

For further details, see supplementary methods and tables (Table S1, Table S2).

Spatial proteomics

Spatial proteomics was used to study the spatial organization of ECM proteins. Liver tissue was stained with H&E. The stained slides were scanned and areas of interest were selected (perilobular and intralobular fibrosis). The slides were then dismounted, the resin was removed, and the tissue was rehydrated. Selected areas were digested using a piezo chemical inkjet printer. The digested areas were then subjected to liquid junction peptide extraction before separation with nano liquid chromatography[7] coupled to a Thermo Scientific Q Exactive Orbitrap mass spectrometer. All mass spectrometry data were processed with MaxQuant (Version 1.6.10.43) using the Andromeda search engine. The proteins were identified using the Decoy version of the complete proteome for reviewed *Homo sapiens* in the UniProt database (release May 2023, 20,422 entries) combined with 262 commonly detected contaminants. The identified proteins were analyzed using Perseus software (http://maxquant.net/perseus/) (version 1.6.10.43). Only proteins that were significant on *t* test analysis were used (p <0.05). Hierarchical clustering and profile plots of statistically significant proteins were performed and visualized by Perseus.

Statistical analysis

Data are expressed as the mean ± SD or the median. A Mann-Whitney *U* test was used for all comparisons. Statistical analyses were performed using Graphpad Prism software (version 5.0, San Diego, CA, USA). p <0.05 was considered to be statistically significant.

Results

Intralobular fibrosis is a key feature of AH in patients with ARLD

The specific patterns of fibrosis in AH and their comparison with Cirrh were investigated with Sirius Red staining and immunohistochemistry of total livers using different markers of ECM or activated myofibroblasts (αSMA, collagen 1 (COL1)-A1, and platelet-derived growth factor receptor alpha (PDGFRα)), which showed that intralobular fibrosis developed specifically in AH livers (Fig. 1A). Co-staining experiments in AH showed intralobular fibrosis marked by αSMA surrounding altered hepatocytes with reduced albumin staining and intense YAP-positive nuclei, suggesting interactions between the mesenchymal and epithelial compartments (Fig. 1B). These data are in line with previous findings showing the marked overexpression of YAP and its target genes in AH livers compared with Cirrh and CTRL.[16]

In addition, bulk proteomic analysis (Fig. 2A) showed differences in ECM composition between patients with AH and Cirrh, with AH livers displaying a specific ECM protein signature compared with Cirrh and CTRL livers. Markers of activated myofibroblasts (*e.g.* vimentin), fibrillar collagens (*e.g.* COL1A1

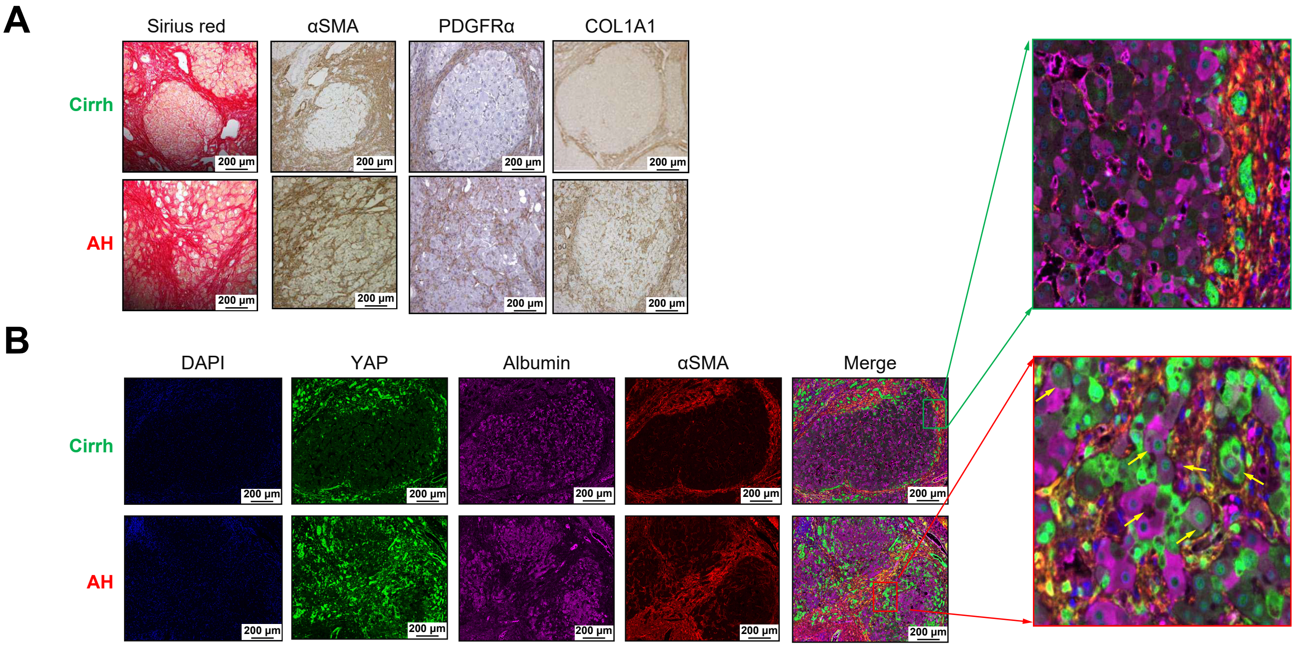

Fig. 1. Intralobular fibrosis is a key feature of AH. (A) Distribution of fibrosis in the liver in AH and *Cirrh.* Representative Sirius red staining of αSMA, PDGFRα, and COL1A1in immunostaining experiments in livers from AH and Cirrh groups; magnification x100. Intense staining was observed in cirrhotic liver samples in fibrotic bands around regenerating nodules. AH liver displayed intense staining throughout the parenchyma. *(B)* Co-immunostaining of YAP (green) and the myofibroblast activation marker, αSMA (red) and albumin (purple) in livers of patients with AH and with Cirrh (magnification x100): in AH livers, intralobular fibrosis was observed marked by αSMA surrounding altered hepatocytes with reduced albumin staining and intense YAP-positive nuclei (examples identified by arrows), suggesting interactions between the mesenchymal and epithelial compartments (magnification x100). αSMA, alpha smooth muscle actin; AH, alcohol-related hepatitis; cirrh, alcohol-related cirrhosis without AH; COL1A1, collagen 1 A1; PDGFRα, platelet-derived growth factor receptor alpha; YAP, Yes-associated protein.

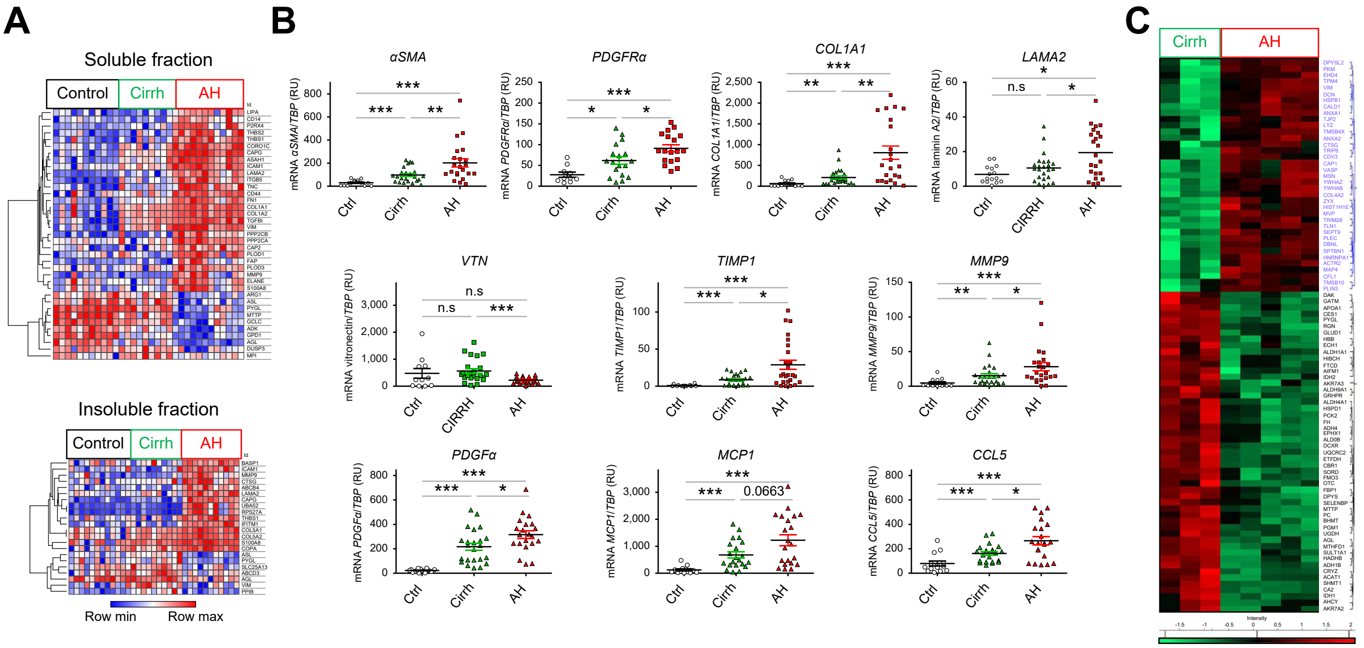

Fig. 2. Specific ECM signature in AH. (A) Bulk proteomic analysis of soluble and insoluble human liver fractions: a heat map comparing expression levels of ECM components in AH liver explants (n = 12), Cirrh liver explants (n = 9) and CTRL liver samples (CTRL, n = 11). The degree of change is the relative variation in protein expression from the center value (*i.e.* mean of all values of that protein in the population); p <0.05 was considered statistically significant. (B) mRNA expression levels of myofibroblast activation markers (*αSMA* and *PDGFRα*), ECM (*COL1A1*, laminin A2, vitronectin, *TIMP1,* and *MMP9*), and chemokines related to fibroblast activation (*PDGFα, MCP1,* and *CCL5*) were compared with the *TBP* housekeeping gene (encoding TATA-binding protein) in CTRL patients (n = 15), patients with AH (n = 22) and patients with Cirrh (n = 24). For clinical data, see Table 1 in the main text; data analyzed with Mann-Whitney U test; *p <0.05; **p <0.01; ***p <0.001. (C) Spatial proteomic analysis showing hierarchical clustering of the most variable proteins between AH (n = 6 areas) and Cirrh (n = 3 areas) extracted from liver intralobular areas. Only proteins that were significant on Student's t test were used (p <0.05). αSMA, alpha smooth muscle actin; AH, alcohol-related hepatitis; CCL5, chemokine ligand 5; Cirrh, alcohol-related cirrhosis without AH; COL1A1, collagen 1 A1; CTRL, control; ECM, extracellular matrix; MCP1, monocyte chemoattractant protein 1; MMP9, matrix metalloproteinase 9; PDGFRα, platelet-derived growth factor receptor alpha; PDGFα, platelet-derived growth factor alpha; TIMP1, tissue inhibitor of metalloproteinase.

and COL1A2), and glycoproteins, such as laminin (LAM)-A2, tenascin, and transforming growth factor (TGF)-β, as well as ECM regulators, such as matrix metalloproteinase-9 (MMP9), cathepsin G, and elastase, were overexpressed in AH livers. Proteins involved in metabolic pathways, such as argino-succinate lyase (ASL) and microsomal triglyceride transfer protein (MTTP), were underexpressed in AH compared with Cirrh livers.

The mRNA expression levels of myofibroblast activation markers αSMA (*ACTA2*) (p <0.01) and *PDGFRα* (p <0.05) as well as other ECM components, such as *LAMA2* (p <0.05), *COL1A1* (p <0.01), ECM-modifying enzymes *MMP9* (p <0.05) and tissue inhibitor of metalloproteinase 1 (*TIMP1*; p <0.05) and the chemokines platelet-derived growth factor alpha (*PDGFα*; p <0.05), chemokine ligand 5 (*CCL5;* p <0.05), and monocyte chemoattractant protein 1 (*MCP1*; p = 0.06) were higher in AH livers than in Cirrh livers (Fig. 2B). Unexpectedly, mRNA expression of vitronectin (*VTN*) was lower (p <0.001) in AH liver samples than in Cirrh liver samples and CTRL livers. By contrast, mRNA expression of the different markers described above (except *VTN*) was higher (all p <0.01) in AH liver samples compared with CTRL liver samples (Fig. 2B).

To compare the distribution of fibrosis in AH *vs.* Cirrh, we performed spatial proteomics in the two groups of patients. The heatmap showed that the spatial distribution of intra-lobular and perilobular fibrosis differed between patients with AH *vs.* those with Cirrh. Spatial proteomics identified two clusters of differentially expressed proteins in the intralobular and perilobular areas of AH (Fig. 2C; Fig. S1). The main changes in the intralobular areas of AH livers involved over-expression of ECM-core proteins (vimentin, COL4A2, and decorin) and ECM-affiliated regulators, such as annexin A1, annexin A2, and cathepsin. Conversely, proteins involved in metabolic pathways controlled by the liver were underex-pressed in AH livers.

The results of these complementary approaches suggest a specific fibrosis profile in AH livers with a specific distribution of fibrosis and differences in ECM composition throughout the liver.

Human *ex vivo* 3D co-culture model demonstrates that AH hepatocytes induce activation of myofibroblasts derived from cirrhotic liver

To explore the role of AH hepatocytes in myofibroblast activation, we developed a 3D co-culture model using AH and Cirrh organoids. After culture in expansion and differentiation medium (Fig. 3A), AH and Cirrh organoids, which varied in size from a few hundred micrometers to several hundred micrometers, contained mature hepatocytes as evaluated by CYP3A4 activity (Fig. S2). As expected, CYP3A4 activity was lower in AH organoids than in Cirrh organoids. AH and Cirrh organoids also had phenotypic characteristics preserved from their native livers. Immunostaining (Fig. 3A) and PCR (Fig. 3B) showed increased expression of cholangiocyte markers (*e.g.* cytokeratin (CK)-19) and decreased expression of hepatocyte

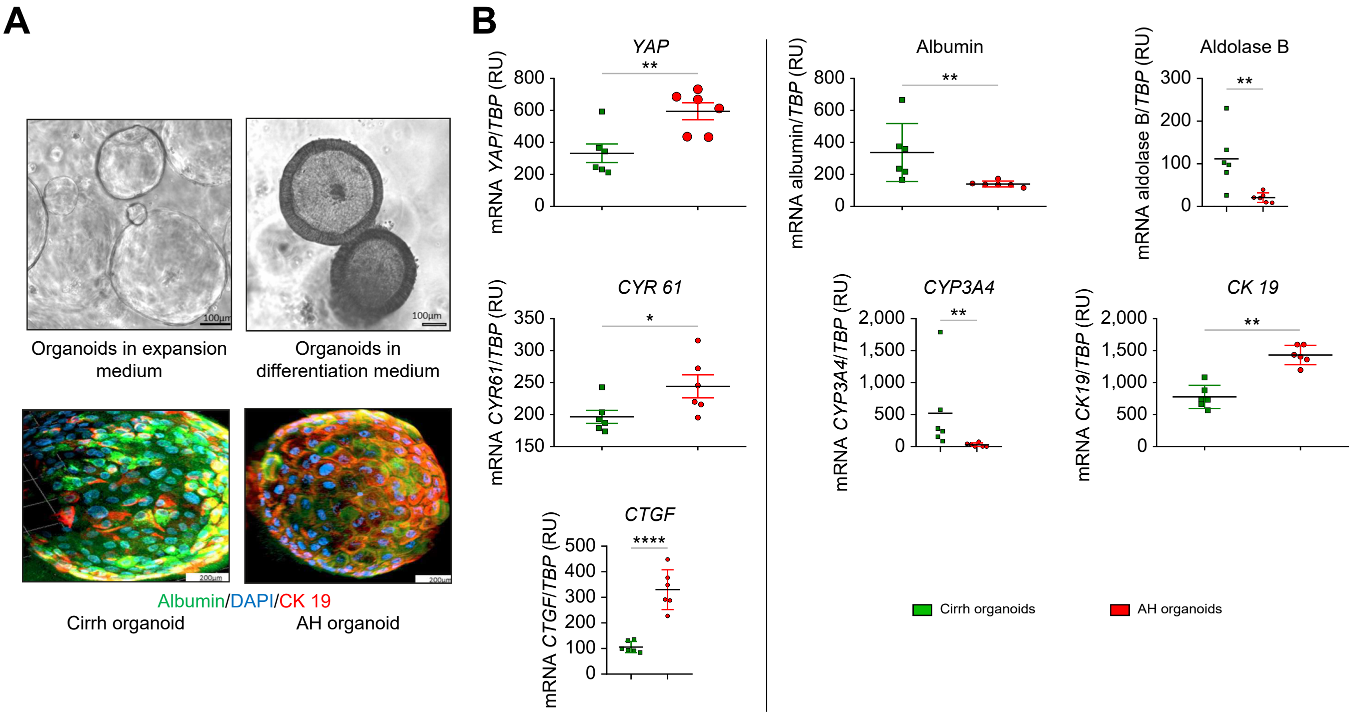

Fig. 3. Characterization and comparative analysis of organoids obtained from AH *vs.* Cirrh livers. (*A*) Organoids cultured in expansion and differentiation medium (top). Immunostaining (marked by the hepatocyte marker albumin in green, cholangiocyte marker CK19 in red) of Cirrh and AH organoids using confocal microscopy; the nuclei are in blue (DAPI) (bottom). (B) Real-time PCRs on organoids from patients with AH (red points, n = 6) and from patients with Cirrh (green triangle, n = 6). Scatter plots showing mRNA expression levels of *YAP* and its target genes *CYR61 and CTGF*, the hepatocyte markers albumin,*Aldolase B and CYP3A4* and the cholangiocyte marker *CK19*. *TBP* (encoding TATA-binding protein) was used as *the* housekeeping gene. Data analyzed with the Mann-Whitney *U* test; ***p <0.05; **p <0.01; ***p <0.001*. AH, alcohol-related hepatitis; Cirrh, alcohol-related cirrhosis without AH; CK19, cytokeratin 19; CTGF: Connective tissue growth factor; CYR61, cellular communication network factor 1; CYP3A4:cytochrome 3A4; YAP, Yes-associated protein.

markers (*e.g.* albumin, CYP3A4, and aldolase B; Fig. 3B) in AH organoids compared with Cirrh organoids**.** mRNA expression of *YAP* (*p* <0.01) and its target genes encoding cellular communication network factor 1 (*CYR61*; *p* <0.01) and connective tissue growth factor (*CTGF*; *p* <0.001) were also higher in AH organoids than in Cirrh organoids (Fig. 3B). Thus, co-culturing organoids with myofibroblasts enables a better understanding of their influence on the activation of the latter; therefore, we developed a 3D co-culture model of organoids and myofibroblasts (Fig. 4A). As expected, mRNA expression of *αSMA* and *PDGFRα* was barely detectable in AH and Cirrh organoids cultured without myofibroblasts (used as a negative control).

In the 3D co-culture model, mRNA expression of *αSMA* (*p* <0.001) and *PDGFRα* (*p* <0.001), *LAMA2* (*p* <0.001), *COL1A1* (*p* <0.001) and the ECM-modifying enzyme *TIMP1* (*p* <0.05) (Fig. 4B) as well as chemokines *PDGFα* (*p* <0.01), *CCL5* (*p* <0.01), and *MCP1* (*p* <0.001) (Fig. 4C) were induced in myofibroblasts co-cultured with AH organoids compared with those co-cultured with Cirrh organoids. In support of *in vivo* data in AH livers, *VTN* expression was lower in AH organoids co-cultured with myofibroblasts than in Cirrh organoids co-cultured with myofibroblasts (Fig. 4B). Furthermore, CFSE labeling and flow cytometry confirmed greater proliferation of myofibroblasts (as shown by lower CFSE intensity) co-cultured with AH organoids than of those co-cultured with Cirrh organoids (Fig. 4D).

In conclusion, when cultured together, AH organoids induced greater activation of myofibroblasts compared with Cirrh organoids, highlighting the role of AH-derived hepatocyte signals in driving fibrogenesis.

YAP activation in hepatocytes induces myofibroblast hyperactivation in AH

To mimic aberrant YAP activation in altered hepatocytes as observed in AH, we generated Cirrh organoids transduced with the constitutively active human transgene *YAP*^{S127A} (YAP-transduced Cirrh organoids). We co-cultured these with myofibroblasts in a 3D co-culture model and compared their impact to that of non-transduced Cirrh organoids.

In YAP-transduced Cirrh organoids, effective transduction was confirmed by greater mRNA expression of the Tag *YAP*^{S127A} (*p* <0.05), *YAP* transgene (*p* <0.01) and its target genes *CYR61*, *NUAK2* (*p* <0.05) and *ANKRD1* (*p* = 0.06) (Fig. S3).

mRNA expression of *αSMA* (*p* <0.05), *PDGFRα* (*p* <0.05), *LAMA2* (*p* <0.05), *COL1A1* (*p* <0.01), *TIMP1* (*p* <0.01) (Fig. 5A), *PDGFα* (*p* <0.05), *CCL5* (*p* = 0.056), and *MCP1* (*p* <0.05) (Fig. 5B) was higher in myofibroblasts co-cultured with YAP-transduced Cirrh organoids. Conversely, as observed *in vivo* and in 3D co-culture with AH organoids, VTN expression was reduced (*p* <0.01), supporting the inhibitory effect of YAP on the VTN regulatory pathway (Fig. 5A). Furthermore, using CFSE

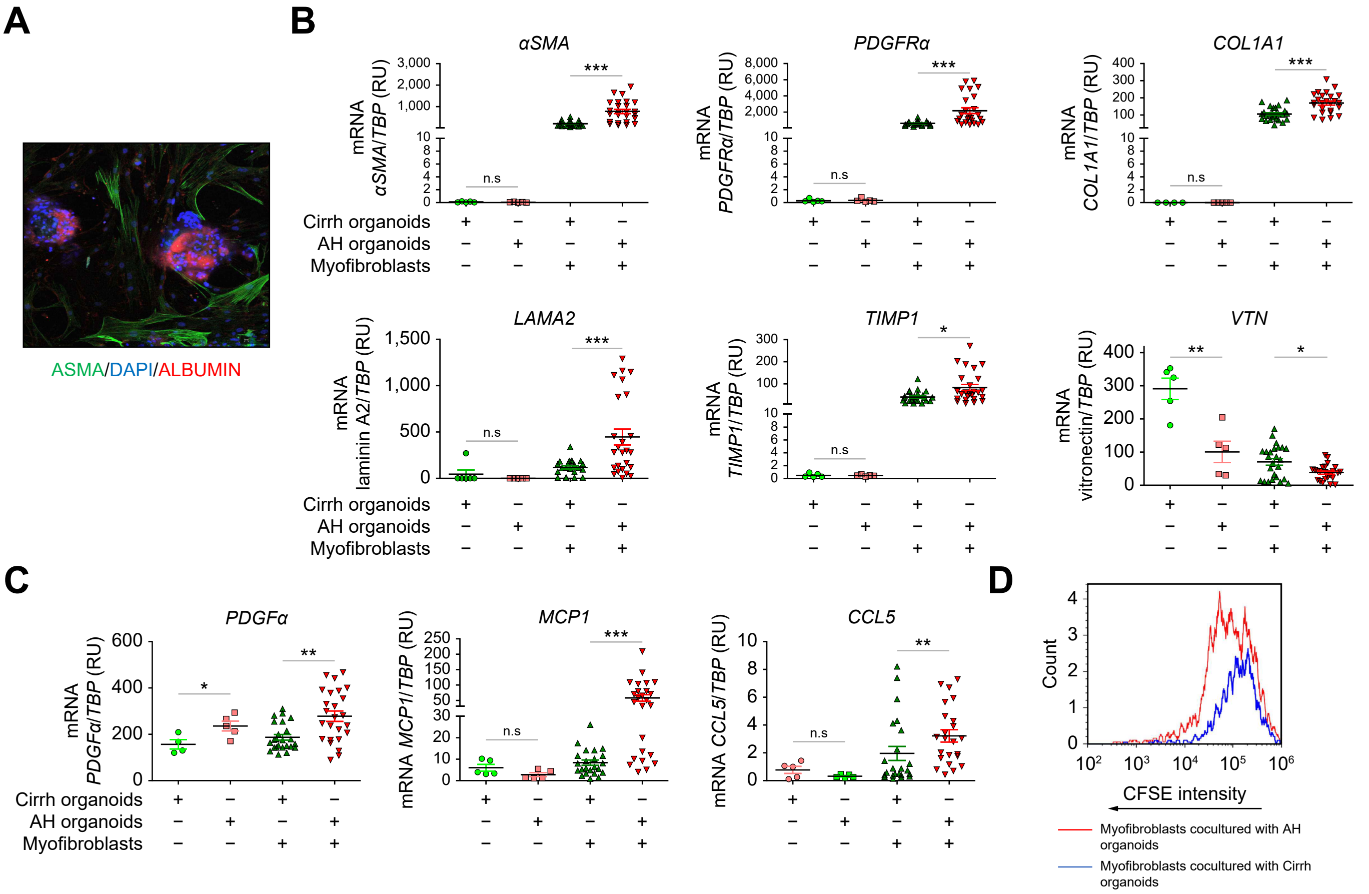

Fig. 4. 3D co-culture of myofibroblasts (derived from cirrhotic liver) with AH or Cirrh organoids. (A) Co-immunostaining of liver organoids (marked by the hepatocyte marker albumin in red) and myofibroblasts (marked by the myofibroblast marker αSMA in green) using confocal microscopy; the nuclei are in blue (DAPI). (B) mRNA expression levels of myofibroblast activation markers (*αSMA* and *PDGFRα*) and ECM markers (*COL1A1, TIMP1*, laminin A2, and vitronectin) were compared with the *TBP* housekeeping gene (encoding TATA-binding protein) in myofibroblasts co-cultured with AH (n = 5, five replicates for each patient) or Cirrh organoids (n = 5, five replicates for each patient). (C) mRNA expression levels of chemokines related to myofibroblast activation (*PDGFα, MCP1*, and *CCL5*) were compared with *TBP* in myofibroblasts co-cultured with AH (n = 5, five replicates for each patient) or Cirrh organoids (n = 5, five replicates for each patient). (D) CFSE intensity in myofibroblasts co-cultured with AH organoids (median: 74,839) was lower than that in the same myofibroblasts co-cultured with Cirrh organoids (median: 123,538), confirming greater proliferation of the former. αSMA, alpha smooth muscle actin; AH, alcohol-related hepatitis; CCL5, chemokine ligand 5; Cirrh, alcohol-related cirrhosis without AH; COL1A1, collagen 1 AI; ECM, extracellular matrix; MCP1, monocyte chemoattractant protein 1; PDGFα, platelet-derived growth factor alpha; PDGFRα, platelet-derived growth factor receptor alpha; TIMP1 tissue inhibitor of metalloproteinase 1.

labeling and flow cytometry, we confirmed a higher proliferation of myofibroblasts (as shown by lower CFSE intensity) co-cultured with YAP-transduced Cirrh organoids than in those co-cultured with non-transduced Cirrh organoids (Fig. 5C).

Thus, myofibroblast activation and proliferation was greater in myofibroblasts co-cultured with YAP-transduced Cirrh organoids than in those co-cultured with non-transduced Cirrh organoids. This suggests that YAP activation in hepatocytes regulates myofibroblast activation.

GDF15 is involved in the paracrine effect of hepatocyte YAP on myofibroblast activation

To evaluate the potential paracrine effect of the aberrant activation of YAP in hepatocytes ('hepatocyte YAP') on the activation of myofibroblasts, we treated myofibroblasts for 48 h in a 2D culture with conditioned media collected from wells containing either YAP^{S127A}-transduced (YAP medium) or non-transduced hepatocytes (CTRL medium). YAP-transduced hepatocytes overexpressed mRNA of *YAP1* (p <0.001) and its target genes *CYR61* (*CCN1*), *TGFb2*, *CTGF* (*CCN2*), *ANKRD1*, and *NUAK2* (all p <0.05) (Fig. S4), confirming the efficiency of the transduction. As previously published by our team,[16] we also observed increased expression of cholangiocyte markers (CK19 and HNF1β) and decreased expression of hepatocyte markers (albumin, CYP3A4, and TAT) (all p <0.05) in our hepatocytes transduced with active YAP.

The expression of *αSMA* (p <0.01), *COL1A1* (p <0.05), *TIMP1* (p <0.05) (Fig. 6A), *PDGFα* (p <0.05), and *MCP1* (p <0.01) was higher in myofibroblasts cultured with YAP medium than in those cultured with CTRL medium (Fig. 6B). YAP medium induced a higher proliferation rate of myofibroblasts compared with CTRL medium-cultured cells, confirmed by nuclear BrdU staining (*i.e.* an increase of 23%; p <0.05) (Fig. 6C). Thus, aberrant YAP activation in hepatocytes induced the activation and proliferation of myofibroblasts through paracrine signals.

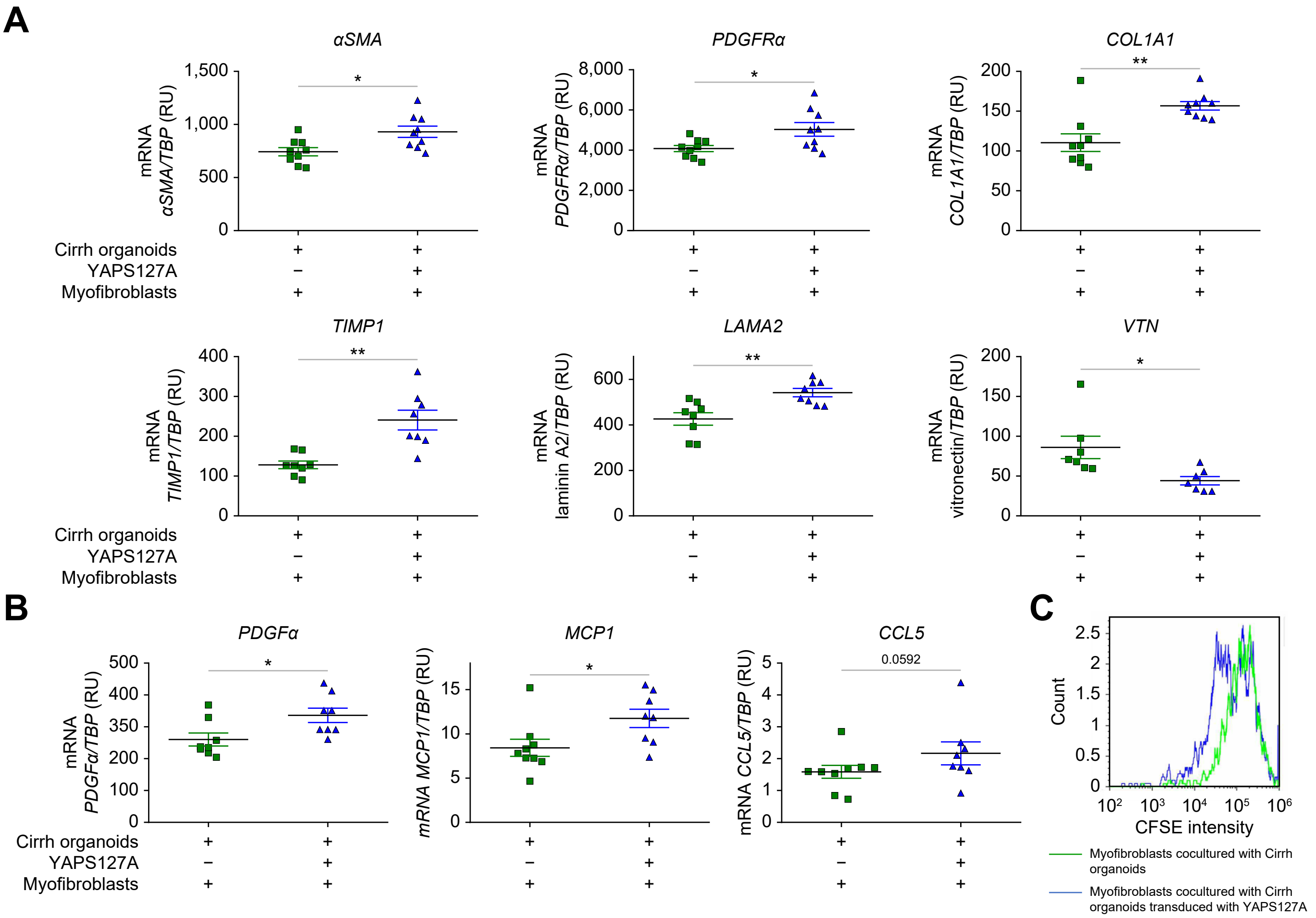

Fig. 5. 3D co-culture of myofibroblasts with transduced or non-transduced Cirrh organoids to mimic YAP aberrant activation during AH. (A) mRNA expression levels of myofibroblast activation markers (*αSMA* and *PDGFRα*) and ECM markers (*COL1A1, TIMP1,* laminin A2, and vitronectin) were compared with the *TBP* housekeeping gene (encoding TATA-binding protein) in myofibroblasts co-cultured with non-transduced (n = 2, four replicates for the first patient and five for the second) or transduced Cirrh organoids (n = 2, four replicates for each patient). (B) mRNA expression levels of chemokines related to myofibroblast activation (*PDGFα, MCP1,* and *CCL5*) were compared with *TBP* in myofibroblasts co-cultured with non-transduced (n = 2, four replicates for the first patient and five for the second) or transduced Cirrh organoids (n = 2, four replicates for each patient). (C) CFSE intensity in myofibroblasts (from Cirrh livers) co-cultured with YAP-transduced Cirrh organoids (median: 65,518) was lower than in the same myofibroblasts co-cultured with Cirrh organoids (median: 123,538), confirming their greater proliferation. αSMA, alpha smooth muscle actin; AH, alcohol-related hepatitis; CCL5, chemokine ligand 5; Cirrh, alcohol-related cirrhosis without AH; COL1A1, collagen 1 AI; ECM, extracellular matrix; MCP1, monocyte chemoattractant protein 1; PDGFα, platelet-derived growth factor alpha; PDGFRα, platelet-derived growth factor receptor alpha; TIMP1 tissue inhibitor of metalloproteinase 1; YAP, yes-associated protein.

Proteomic analysis of conditioned media collected from YAP^{S127A}-transduced and non-transduced hepatocytes was performed. We detected differential expression of a few proteins, such GDF15 (Fig. 7A). Based on literature suggesting its potential involvement in fibroblast activation, we investigated the role of GDF15 in the fibrogenesis observed in AH. AH livers displayed intense GDF15 staining throughout the entire parenchyma, mainly located in hepatocytes (Fig. 7B). Expression of *GDF15* mRNA was higher in the livers of patients with AH compared with those of patients with Cirrh (p <0.05; Fig. 7C). We also observed higher expression of *GDF15* mRNA in *YAP^{S127A}*-transduced hepatocytes compared with in non-transduced hepatocytes (p <0.01; Fig. 7D). Expression of *GDF15* mRNA was also higher in myofibroblasts co-cultured with AH organoids compared with myofibroblasts co-cultured with Cirrh organoids (p <0.01; Fig. 7E). To investigate the effect of GDF15, we treated human primary myofibroblasts with GDF15 for 48 h (at a concentration of 10 ng/ml) and observed a significant increase in the mRNA expression of *αSMA, COL1A1,* and *PDGFα* (all p <0.01) compared with non-GDF15-treated myofibroblasts (Fig. 7F). A significant difference (p <0.05) was also found in PDGF-AB levels between supernatants collected from wells containing myofibroblasts treated or not with GDF 15 (Fig. 7G).

Besides profibrotic signals, such as CYR61, TGFβ2, and CTGF, our findings suggest that GDF15 is involved in the paracrine effect of the aberrant activation of YAP in hepatocytes on myofibroblast activation.

Discussion

The present study showed that AH livers display a specific distribution of fibrosis in intralobular areas. Aberrant YAP activation in hepatocytes colocalized with intralobular fibrosis.

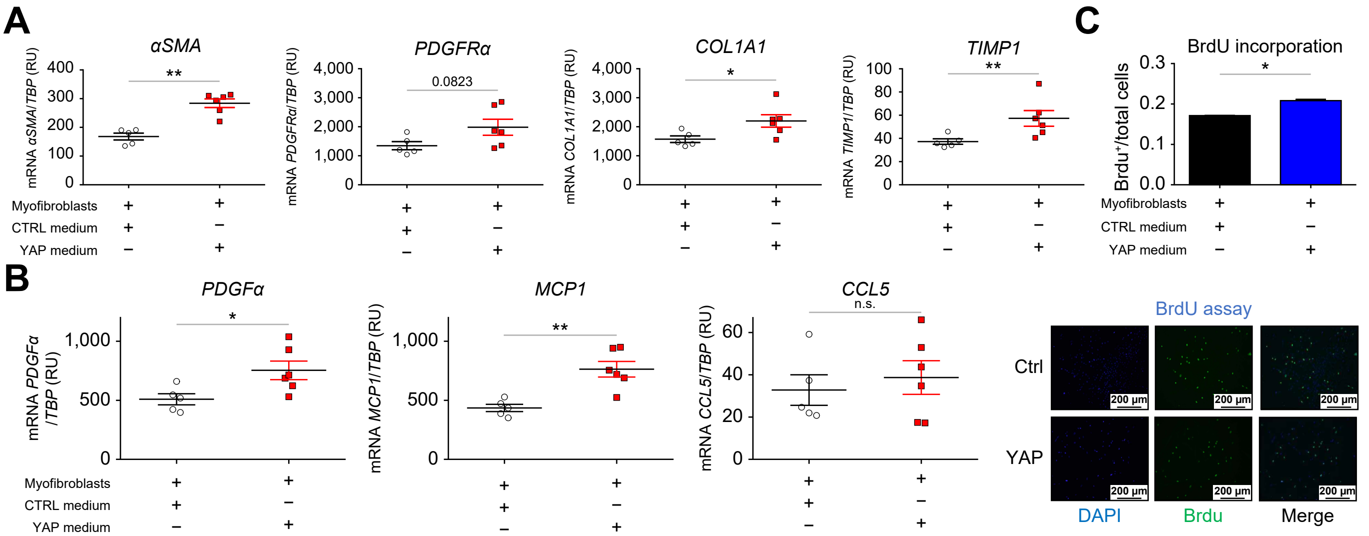

Fig. 6. 2D culture of myofibroblasts with YAP or CTRL-conditioned medium. (A) mRNA expression levels of myofibroblast activation markers (*αSMA* and *PDGFRα*) and ECM markers (*COL1A1* and *TIMP1*) were compared with the *TBP* housekeeping gene (encoding TATA-binding protein) in myofibroblasts cultured with CTRL (n = 5 samples) or YAP medium (n = 6 samples). (B) mRNA expression levels of chemokines related to myofibroblast activation (*PDGFα, MCP1,* and *CCL5*) were compared with *TBP* in myofibroblasts cultured with CTRL (n = 5 samples) or YAP medium (n = 6 samples). Data analyzed by Mann-Whitney *U* test; **p* <0.05; ***p* <0.01; ****p* <0.001. (C) Immunostaining evaluating BrdU incorporation 48 h after human primary myofibroblasts were cultured with YAP or CTRL medium. Using Cell discovery 4, YAP medium induced a higher proliferation rate of myofibroblasts compared with CTRL medium-cultured cells, confirmed by nuclear BrdU staining (*i.e.* an increase of 23%, *p* <0.05). αSMA, alpha smooth muscle actin; CCL5, chemokine ligand 5; COL1A1, collagen 1 AI; CTRL, control; ECM, extracellular matrix; MCP1, monocyte chemoattractant protein 1; PDGFα, platelet-derived growth factor alpha; PDGFRα, platelet-derived growth factor receptor alpha; TIMP1 tissue inhibitor of metalloproteinase 1; YAP, yes-associated protein.

Interestingly, this distribution and colocalization were associated with a specific bulk and spatial ECM protein signature. We originally developed liver organoids from patients with severe AH or decompensated cirrhosis. AH organoids overexpressed YAP, activating myofibroblast expression of ECM components and stimulating myofibroblast proliferation. After transducing Cirrh organoids with a constitutively active YAP, these effects were mimicked. A 2D culture model of myofibroblasts with conditioned media collected from YAP-transduced hepatocytes showed that these effects were related, in part, to a paracrine effect mediated by GDF15. The level of VTN expression was lower in AH livers and myofibroblasts co-cultured with AH organoids. In previous studies,[26,27] VTN has been shown to be a marker of mature fibrosis. Thus, our hypothesis is that intralobular fibrosis is less mature in AH livers.

We assessed whether the altered epithelial compartment found in AH influenced fibrogenesis in AH by developing a 3D *ex vivo* co-culture model of myofibroblasts and AH or Cirrh organoids. To our knowledge, this is the first study to use this model. Activation of myofibroblasts co-cultured with AH organoids was increased, suggesting that the altered hepatocytes influence the specific fibrosis profile observed in AH.

Hyun *et al.*[28] observed that the accumulation of fetal isoforms of Hippo kinases during AH leads to activation of YAP/TAZ. More recently, our group found that dysregulation of the Hippo/YAP pathway is involved in the ineffective hepatocyte regeneration observed in AH.[16] Mannaerts *et al.*[18] and, more recently, Du *et al.*[29] demonstrated the key role of YAP activation in the activation of HSCs, with knockout or inhibition of YAP preventing HSC activation.[18,29] Activation of YAP in Cirrh organoids induced changes in myofibroblasts comparable to those observed in AH organoids, suggesting that aberrant YAP activation in hepatocytes has a role in the activation of myofibroblasts observed in AH. To our knowledge, this is the first time that the impact of aberrant YAP activation in hepatocytes on the activation of myofibroblasts has been demonstrated in AH. Machado *et al.* observed that the accumulation of duct cells with activated YAP in patients with metabolic dysfunction-associated steatohepatitis (MASH)[30] parallels myofibroblast accumulation and fibrosis progression. Mooring *et al.* used several models of liver injury (*e.g.* chronic CCl_4 administration or a CDE-supplement diet) to observe that increased hepatocyte levels and activity of YAP/TAZ were associated with the development of liver inflammation and fibrosis. By contrast, mice with hepatocyte-specific YAP and YAP/TAZ knockout exhibited limited myofibroblast expansion, less inflammation, and decreased fibrosis after CCl_4 injury.[17] However, conclusions of a recent study on the role of YAP in fibrogenesis[31] were contradictory. In a model of ischemia–reperfusion injury, the authors observed that the formation of fibrosis was diminished during the recovery phase in mice pretreated with a YAP activator. By contrast, mice pretreated with a YAP inhibitor developed severe fibrosis.[31] However, in a follow-up letter to the editor, the same team published new results of their ongoing studies showing that YAP activation 1 h after 90 min of ischemia and 6 h of reperfusion was associated with increased HSC activation, resulting in severe fibrosis 7 days later.[32] Thus, the role of aberrant YAP activation in hepatocytes on the activation of myofibroblasts appears to be complex. Our study provides new evidence for an indirect role of increased YAP levels in hepatocytes on fibrogenesis in AH.

We attempted to identify mechanisms involved in the role of YAP hyperactivation in hepatocytes on myofibroblast activation. Mannaerts *et al.*[18] found marked upregulation of ANKRD1

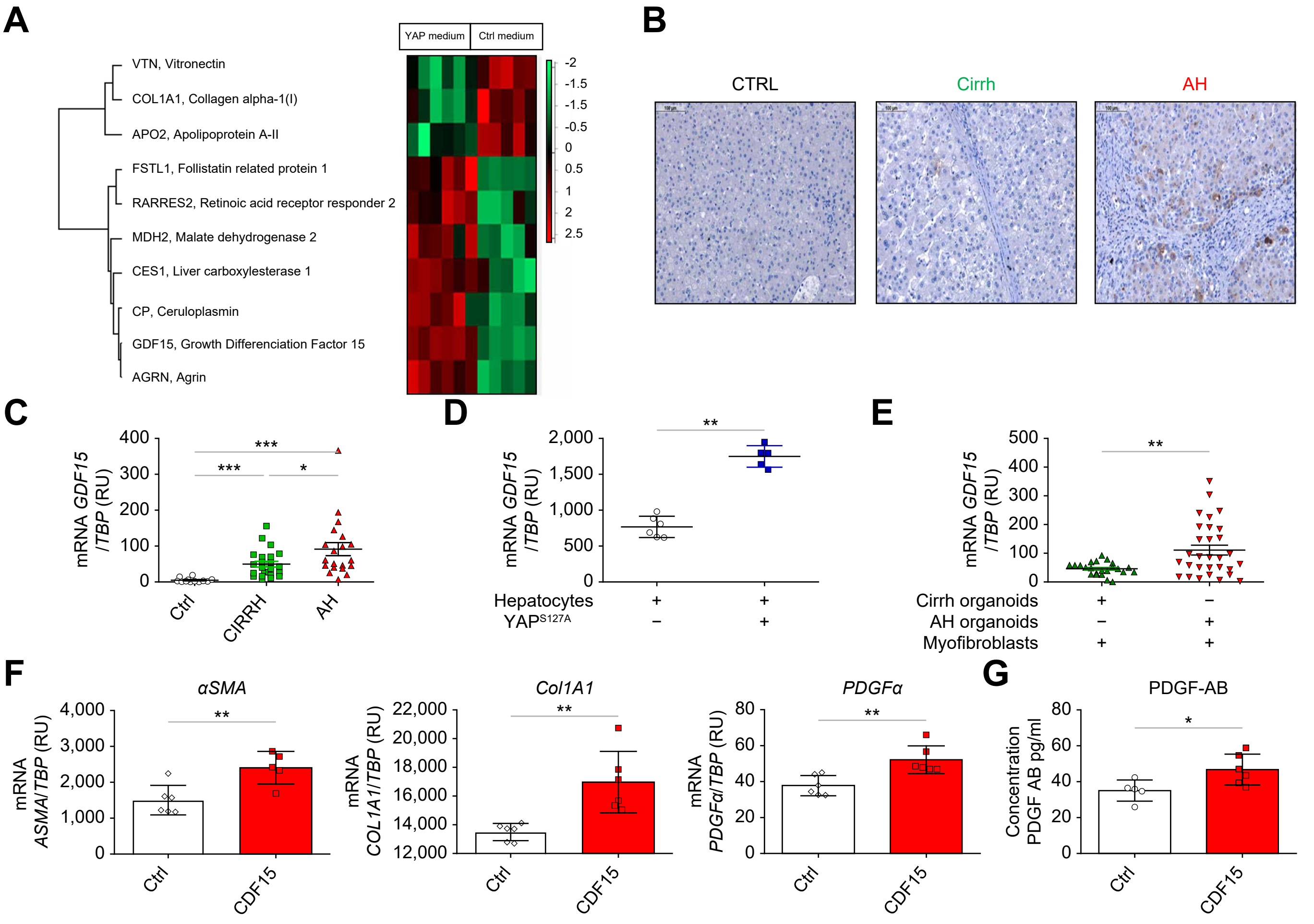

Fig. 7. Paracrine effect of YAP activation in hepatocytes on myofibroblast activation: role of GDF15. (A) Proteomic analysis of YAP-transduced (n = 6 samples) and non-transduced hepatocyte (CTRL)-conditioned media (n = 5 samples). The degree of change is the relative variation in protein expression compared with the center value (*i.e.* mean of all values of that protein in the population); p <0.05 was considered statistically significant. (B) Representative GDF15 immunostaining experiments in livers from control (CTRL), Cirrh, and AH groups (magnification x100). AH livers displayed intense staining throughout the parenchyma mainly located in hepatocytes. (C) mRNA expression levels of *GDF15* compared with the *TBP* housekeeping gene (encoding TATA-binding protein) in CTRL patients (n = 15), patients with AH (n = 22) and patients with Cirrh (n = 24). For clinical data, see Table 1 in the main text. (D) mRNA expression levels of *GDF15* compared with *TBP* in YAP^{S127A}-transduced hepatocytes compared with that of non-transduced hepatocytes. (E) mRNA expression levels of *GDF15* compared with *TBP* in myofibroblasts co-cultured with AH and Cirrh organoids. (F) mRNA expression levels of *αSMA, COL1A1,* and *PDGFα* were compared with *TBP* in myofibroblasts treated or not with GDF15 (10 ng/ml) (n = 5 replicates per condition) for 48 h. (G) ELISA: PDGF-AB protein levels in supernatants collected from myofibroblasts treated or not with GDF15 (10 ng/ml) (n = 5 replicates per condition) for 48 h. Data in (C–G) analyzed by Mann-Whitney *U test; *p* <0.05; ***p* <0.01; ****p* <0.001. αSMA, alpha smooth muscle actin; AH, alcohol-related hepatitis; Cirrh, alcohol-related cirrhosis without AH; COL1A1, collagen 1 AI; CTRL, control; GDF15, growth differentiation factor 15; PDGFα, platelet-derived growth factor alpha; YAP, yes-associated protein.

as well as CTGF in parallel to YAP activation. CTGF and TGFβ2 were overexpressed in transduced human hepatocytes with a constitutively active YAP. Moreover, conditioned medium collected from wells containing those hepatocytes induced the activation and proliferation of myofibroblasts. CTGF overexpression has been identified in human liver fibrosis and activated myofibroblasts,[33,34] while TGFβ is a master regulator of fibrosis through both canonical and non-canonical pathways.[35] We also observed increased expression of CYR61 in transduced Cirrh organoids and in transduced human hepatocytes involved in myofibroblast activation. Mooring *et al.* showed that CYR61 expression was correlated with the level of YAP/TAZ in hepatocytes of patients with MASH. CYR61 inhibition in mice was also associated with a significant reduction in fibrosis.[17] Furthermore, proteomic analysis of CTRL and YAP medium found increased expression of GDF15 in YAP medium and treatment with GDF15 increased myofibroblast activation. GDF15 expression correlated with the severity of fibrosis in patients with chronic liver disease[36–39] and was associated with an increased risk of liver cancer.[40–42] In *ex vivo* models based on cell lines (Huh7, LX2 and HepG2), the increase in GDF15 was correlated with activation of these cell lines and production of type 1 collagen via activating ERK1/2 and Smad3 signaling.[43] *In vivo*, injection of antibodies against GDF15 led to a reduction of fibrosis in mouse models in which liver fibrosis was induced.[44] Taken together, these data suggest that aberrant YAP activation in hepatocytes induces myofibroblast activation and infiltration of fibrosis via different mechanisms related to the expression of soluble proteins or chemokines, including GDF15.

In conclusion, the present study shows that the intralobular distribution of fibrosis is specific in AH associated with a defined protein signature. Results from co-cultured AH and YAP-transduced Cirrh organoids with myofibroblasts showed that YAP has a role in the interplay between altered hepatocytes and myofibroblasts in the specific fibrogenesis observed in AH. Thus, targeting YAP could be a promising strategy to improve regeneration and reduce fibrogenesis in AH livers and, therefore, its associated complications. However, further preclinical studies should evaluate the beneficial effects of YAP interference in ARLD.

Affiliations

[1]*Univeristy of Lille, Inserm, CHU Lille, U1286 - INFINITE - Institute for Translational Research in Inflammation, F-59000 Lille, France;* [2]*CHU Lille, Service des MALADIES de l'Appareil Digestif, F-59000 Lille, France;* [3]*Univeristy of Lille, CNRS, Inserm, CHU Lille, UMR9020-U1277 - CANTHER – Cancer Heterogeneity Plasticity and Resistance to Therapies, F-59000 Lille, France;* [4]*CHU Lille, Service de Chirurgie Digestive et Transplantations, F-59000 Lille, France;* [5]*Univeristy of Lille, CNRS, UAR 3290 - MSAP - Miniaturisation pour la Synthèse, l'Analyse et la Protéomique, F-59000 Lille, France;* [6]*Univeristy of Lille, Inserm, CHU Lille, U1189 - ONCO-THAI - Assisted Laser Therapy and Immunotherapy for Oncology, F-59000 Lille, France;* [7]*U1011-EGID, Institut Pasteur de Lille, CHU Lille, Inserm, Univ. Lille, F-59000 Lille, France;* [8]*CHU Lille, Service d'Anatomopathologie, F-59000 Lille, France*

Abbreviations

αSMA, alpha smooth muscle actin; AH, alcohol-related hepatitis; ARLD, alcohol-related liver disease; ASL, arginosuccinate lyase; AAV, adeno-associated virus; BME, basement membrane extract type; CCL5, chemokine ligand 5; cirrh, alcohol-related cirrhosis without AH; CK, cytokeratin; COL1, collagen 1; CRP, C reactive protein; CTGF, connective tissue growth factor; CTRL, control; CYR61, cellular communication network factor 1; ECM, extracellular matrix; GDF15, growth differentiation factor 15; HSC, hepatic stellate cells; FACS, fluorescence-activated cell sorting; INR, international normalized ratio; LAM, laminin; LC, liquid chromatography; MASH, metabolic dysfunction-associated steatohepatitis; MS, mass spectrometry; MCP1, monocyte chemoattractant protein 1; MELD, model for end-stage liver disease; MMP9, matrix metalloproteinase 9; MTTP, microsomal triglyceride transfer protein; PCF, pericellular fibrosis; PDGFRα, platelet-derived growth factor receptor alpha; PDGFα, platelet-derived growth factor alpha; TGF, transforming growth factor; TIMP1, tissue inhibitor of metalloproteinase 1; YAP, yes-associated protein; VTN, vitronectin.

Conflict of interest

The authors of this study declare that they do not have any conflict of interest.

Please refer to the accompanying ICMJE disclosure forms for further details.

Financial support

The authors acknowledge funding from the Association Française pour l'Etude du Foie, Institut National de la Santé et de la Recherche Médicale (INSERM), CHU de Lille and Région Hauts de France, from the Agence Nationale de la Recherche (ANR) ANR-21-CE17-0016-01 and ANR-21-CE14-0032-02. Work at U1286 – INFINITE and INSERM U1011 was supported by grants from the Fondation pour la Recherche Médicale (Equipes Labellisées: DEQ20150331724 and EQU202003010299). The UAR 3290 (MSAP) platform acknowledges the IBiSA Network for financial support. Work by CS and P-JD was supported by the 'Métropole Européenne de Lille' (MEL N°Convention_2021_ESR_11) and Agence Nationale pour la Recherche (ANR CPJ_Sobolewski).

Authors' contributions

Design of the study: LCNW, MBS, PM, LD, AL. Acquisition of data: LCNW, MBS, CS, MEA, FB, EB, FM, ST, CR, P-JD, FM, SD, VG, LD, AL. Statistical analysis: LCNW, LD, AL. Drafting of the manuscript and critical review: LCNW, PM, LD, AL. Funding searching: LCNW, JE, PM, LD, AL.

Data availability

Proteomics and spatial proteomics data are being published and will be made publically available shortly.

Acknowledgements

The authors thank Fatima Hariss and Tom Rousset for their technical support. We also thank the immunohistochemistry laboratory at the Center for Biology-Pathology (Lille University Medical Center), especially Nicolas Van Pouck for his technical support. We are grateful to the Centre de Production de Vecteurs facility (INSERM U1089, Nantes, France) for production of the AAVs. We also thank Antonino Bongiovanni and Meryem Tardivel from the BioImaging Center Lille (F-59000 Lille, France) for the access and excellent technical support. The authors would like to thank the PRISM laboratory (INSERM U1192, Lille, France) especially Soulaimane Aboulouard for his excellent technical support.

Supplementary data

Supplementary data to this article can be found online at https://doi.org/10.1016/j.jhepr.2025.101580.

References

Author names in bold designate shared co-first authorship

[1] Mathurin P, Moreno C, Samuel D, et al. Early liver transplantation for severe alcoholic hepatitis. N Engl J Med 2011;365:1790–1800.
[2] Germani G, Angrisani D, Addolorato G, et al. Liver transplantation for severe alcoholic hepatitis: a multicenter Italian study. Am J Transpl 2022;22:1191–1200.
[3] Im GY, Kim-Schluger L, Shenoy A, et al. Early liver transplantation for severe alcoholic hepatitis in the United States–a single-center experience. Am J Transpl 2016;16:841–849.
[4] Lee BP, Mehta N, Platt L, et al. Outcomes of early liver transplantation for patients with severe alcoholic hepatitis. Gastroenterology 2018;155:422–430.
[5] **Louvet A**, Thursz MR, Kim DJ, et al. Corticosteroids reduce risk of death within 28 days for patients with severe alcoholic hepatitis, compared with pentoxifylline or placebo-a meta-analysis of individual data from controlled trials. Gastroenterology 2018;155:458–468.
[6] Rattan P, Shah VH. Review article: current and emerging therapies for acute alcohol-associated hepatitis. Aliment Pharmacol Ther 2022;56:28–40.
[7] Gawrieh S, Dasarathy S, Tu W, et al. Randomized trial of anakinra plus zinc vs. prednisone for severe alcohol-associated hepatitis. J Hepatol 2024;80:684–693.
[8] Szabo G, Mitchell M, McClain CJ, et al. IL-1 receptor antagonist plus pentoxifylline and zinc for severe alcohol-associated hepatitis. Hepatology 2022;76:1058–1068.
[9] Lackner C, Stauber RE, Davies S, et al. Development and prognostic relevance of a histologic grading and staging system for alcohol-related liver disease. J Hepatol 2021;75:810–819.
[10] Lackner C, Tiniakos D. Fibrosis and alcohol-related liver disease. J Hepatol 2019;70:294–304.
[11] Tsuchida T, Friedman SL. Mechanisms of hepatic stellate cell activation. Nat Rev Gastroenterol Hepatol 2017;14:397–411.
[12] **Louvet A**, **Mathurin P**. Alcoholic liver disease: mechanisms of injury and targeted treatment. Nat Rev Gastroenterol Hepatol 2015;12:231–242.
[13] **Dubuquoy L**, **Louvet A**, Lassailly G, et al. Progenitor cell expansion and impaired hepatocyte regeneration in explanted livers from alcoholic hepatitis. Gut 2015;64:1949–1960.
[14] Espanol-Suner R, Carpentier R, Van Hul N, et al. Liver progenitor cells yield functional hepatocytes in response to chronic liver injury in mice. Gastroenterology 2012;143:1564–1575.
[15] Lorenzini S, Bird TG, Boulter L, et al. Characterisation of a stereotypical cellular and extracellular adult liver progenitor cell niche in rodents and diseased human liver. Gut 2010;59:645–654.
[16] Bou Saleh M, Louvet A, Ntandja-Wandji LC, et al. Loss of hepatocyte identity following aberrant YAP activation: a key mechanism in alcoholic hepatitis. J Hepatol 2021;75:912–923.
[17] Mooring M, Fowl BH, Lum SZC, et al. Hepatocyte stress increases expression of yes-associated protein and transcriptional coactivator with PDZ-binding motif in hepatocytes to promote parenchymal inflammation and fibrosis. Hepatology 2020;71:1813–1830.

[18] Mannaerts I, Leite SB, Verhulst S, et al. The Hippo pathway effector YAP controls mouse hepatic stellate cell activation. J Hepatol 2015;63:679–688.
[19] Jalan-Sakrikar N, Brevini T, Huebert RC, et al. Organoids and regenerative hepatology. Hepatology 2023;77:305–322.
[20] Nuciforo S, Heim MH. Organoids to model liver disease. JHEP Rep 2021;3:100198.
[21] **Louvet A**, Naveau S, Abdelnour M, et al. The Lille model: a new tool for therapeutic strategy in patients with severe alcoholic hepatitis treated with steroids. Hepatology 2007;45:1348–1354.
[22] Broutier L, Andersson-Rolf A, Hindley CJ, et al. Culture and establishment of self-renewing human and mouse adult liver and pancreas 3D organoids and their genetic manipulation. Nat Protoc 2016;11:1724–1743.
[23] Erde J, Loo RR, Loo JA. Enhanced FASP (eFASP) to increase proteome coverage and sample recovery for quantitative proteomic experiments. J Proteome Res 2014;13:1885–1895.
[24] Vivier S, Chepy A, Bray F, et al. Passages in culture and stimulation conditions influence protein expression of primary fibroblasts. Proteomics 2022;22:e2100116.
[25] Tyanova S, Temu T, Sinitcyn P, et al. The Perseus computational platform for comprehensive analysis of (prote)omics data. Nat Methods 2016;13:731–740.
[26] Inuzuka S, Ueno T, Torimura T, et al. Vitronectin in liver disorders: biochemical and immunohistochemical studies. Hepatology 1992;15:629–636.
[27] Koukoulis GK, Shen J, Virtanen I, et al. Vitronectin in the cirrhotic liver: an immunomarker of mature fibrosis. Hum Pathol 2001;32:1356–1362.
[28] Hyun J, Sun Z, Ahmadi AR, et al. Epithelial splicing regulatory protein 2-mediated alternative splicing reprograms hepatocytes in severe alcoholic hepatitis. J Clin Invest 2020;130:2129–2145.
[29] Du K, Maeso-Diaz R, Oh SH, et al. Targeting YAP-mediated HSC death susceptibility and senescence for treatment of liver fibrosis. Hepatology 2023;77:1998–2015.
[30] Machado MV, Michelotti GA, Pereira TA, et al. Accumulation of duct cells with activated YAP parallels fibrosis progression in non-alcoholic fatty liver disease. J Hepatol 2015;63:962–970.
[31] Liu Y, Lu T, Zhang C, et al. Activation of YAP attenuates hepatic damage and fibrosis in liver ischemia-reperfusion injury. J Hepatol 2019;71:719–730.
[32] Liu Y, Xu N, Ji H. Reply to: ‘Myofibroblast YAP/TAZ is dispensable for liver fibrosis in mice’. J Hepatol 2021;75:241–243.
[33] Rachfal AW, Brigstock DR. Connective tissue growth factor (CTGF/CCN2) in hepatic fibrosis. Hepatol Res 2003;26:1–9.
[34] Williams EJ, Gaca MD, Brigstock DR, Arthur MJ, Benyon RC. Increased expression of connective tissue growth factor in fibrotic human liver and in activated hepatic stellate cells. J Hepatol 2000;32:754–761.
[35] Meng XM, Nikolic-Paterson DJ, Lan HY. TGF-beta: the master regulator of fibrosis. Nat Rev Nephrol 2016;12:325–338.
[36] Bilson J, Scorletti E, Bindels LB, et al. Growth differentiation factor-15 and the association between type 2 diabetes and liver fibrosis in NAFLD. Nutr Diabetes 2021;11:32.
[37] Koo BK, Um SH, Seo DS, et al. Growth differentiation factor 15 predicts advanced fibrosis in biopsy-proven non-alcoholic fatty liver disease. Liver Int 2018;38:695–705.
[38] Li Z, Liu Y, Li X, et al. Association between Circulating growth differentiation factor 15 and cirrhotic primary biliary cholangitis. Biomed Res Int 2020;2020:5162541.
[39] Prystupa A, Kicinski P, Luchowska-Kocot D, et al. Association between serum selenium concentrations and levels of proinflammatory and profibrotic cytokines-interleukin-6 and growth differentiation factor-15, in patients with alcoholic liver cirrhosis. Int J Environ Res Public Health 2017;14:437.
[40] Kumazaki S, Hikita H, Tahata Y, et al. Serum growth differentiation factor 15 is a novel biomarker with high predictive capability for liver cancer occurrence in patients with MASLD regardless of liver fibrosis. Aliment Pharmacol Ther 2024;60:327–339.
[41] Chen J, Tang D, Xu C, et al. Evaluation of serum GDF15, AFP, and PIVKA-II as diagnostic markers for HBV-associated hepatocellular carcinoma. Lab Med 2021;52:381–389.
[42] Myojin Y, Hikita H, Tahata Y, et al. Serum growth differentiation factor 15 predicts hepatocellular carcinoma occurrence after hepatitis C virus elimination. Aliment Pharmacol Ther 2022;55:422–433.
[43] Dong G, Ma M, Lin X, et al. Treatment-damaged hepatocellular carcinoma promotes activities of hepatic stellate cells and fibrosis through GDF15. Exp Cell Res 2018;370:468–477.
[44] Qi P, Ma MZ, Kuai JH. Identification of growth differentiation factor 15 as a pro-fibrotic factor in mouse liver fibrosis progression. Int J Exp Pathol 2021;102:148–156.

Keywords: Alcohol-related hepatitis; Alcohol-related cirrhosis; Organoids; Fibrosis; Hippo/YAP; 3D culture; Proteomics.
Received 27 January 2025; received in revised form 20 August 2025; accepted 22 August 2025; Available online 29 August 2025

Journal of Hepatology, Volume *7*

Supplemental information

Alcohol-related hepatitis induces a specific fibrosis profile through YAP activation in myofibroblasts

Line Carolle Ntandja Wandji, Mohamed Bou Saleh, Cyril Sobolewski, Mehdi El Amrani, Fabrice Bray, Emmanuel Boleslawski, Jérôme Eeckhoute, Pierre-Jean Devaux, François Maggiotto, Solenne Taront, Christian Rolando, Viviane Gnemmi, Philippe Mathurin, Laurent Dubuquoy, and Alexandre Louvet

Alcohol-related hepatitis induces a specific fibrosis profile through YAP activation in myofibroblasts

Line Carolle Ntandja Wandji, Mohamed Bousaleh, Cyril Sobolewski, Mehdi El Amrani, Fabrice Bray, Emmanuel Boleslawski, Jérôme Eeckhoute, Pierre-Jean Devaux, François Maggiotto, Solenne Taront, Christian Rolando, Viviane Gnemmi, Philippe Mathurin, Laurent Dubuquoy, Alexandre Louvet

Table of contents

Supplementary methods……………………………………………………………..2

Supplementary figures………………………………………………………………4

Supplementary tables……………………………………………………………...7

Supplementary reference…………………………………………………………..10

Supplementary methods

➢ Isolation of mRNA and real-time PCR

We used a Nucleospin®RNAII kit (Macherey-Nagel, Hoerdt, France) and followed the instructions provided to isolate mRNA from our hepatocytes, myofibroblasts, liver organoids and liver samples. First-strand cDNA was synthesized from 1 µg total RNA, using a High Capacity cDNA Reverse Transcription Kit (Applied Biosystems, Courtaboeuf, France). Real-time PCR was performed using Power SYBR Green PCR Master Mix in a StepOne plus system (both from Applied Biosystems, Foster City, CA, 4 USA). The gene coding for human TATA-binding protein (TBP) was used as housekeeping gene. Specific PCR primers were designed using Primer3 software (https://primer3.org/; **See supplementary Table 1 for details**). The specificity of the amplification and the absence of primer dimerization were ensured by performing a dissociation curve.

➢ Sirius red staining

Deparaffinization and rehydration of the slides were done respectively using the following series of washes: xylene, ethanol and distilled water respectively. Sections were immersed in picro Sirius red solution and stain for 60 min at room temperature. Slides were quickly rinse in acetic acid solution and then dehydrated in absolute alcohol. The slides were mounted under coverslips using resinous mounting medium.

➢ Immunohistochemistry and immunofluorescence

Liver samples fixed with 4% paraformaldehyde were embedded in paraffin. Four-micrometer-thick sections were exposed to primary antibodies overnight (**see Supplementary table 2 for details**) after a specific antigen retrieval step. After washing in Tris buffered saline + 0.05% Tween 20, sections were incubated for 30 min with EnVision+System-HRP Labelled Polymer (Dako Laboratories, Trappes, France) or the EnVision G/2 System/AP Kit for alkaline phosphatase staining. Negative controls were incubated with irrelevant serum or an isotype-matched immunoglobulin instead of the specific antibody. Stained slides were observed under the microscope (Leica, Bensheim, Germany) and analyzed by pathologists.

Organoids cocultured with fibroblasts were embedded in 4% paraformaldehyde without disrupting the BME 2. The same steps above were then realized after permeabilization using Triton 0,01% stored at +4°C.

➢ Proteomic analysis

To identify inflammation/fibrosis-related proteins, significant dysregulated proteins between cirrhosis and AH (adjusted P-value using the Bonferoni-Hochsberg method) were cross-compared with the following human gene sets from the GSEA database (https://www.gsea-msigdb.org/gsea/msigdb/human/genesets.jsp):

HALLMARK_EPITHELIAL_MESENCHYMAL_TRANSITION (M5930), GOBP_FIBROBLAST_MIGRATION (M22676), GOBP_MYOFIBROBLAST_DIFFERENTIATION (M23447), HALLMARK_INFLAMMATORY_RESPONSE (M5932), SA_MMP_CYTOKINE_CONNECTION (M11736), REACTOME_ACTIVATION_OF_MATRIX_METALLOPROTEINASES (M26976), HALLMARK_TGF_BETA_SIGNALING (M5896), GOBP_FIBROBLAST_ACTIVATION (M24593), PLASARI_TGFB1_TARGETS_1HR_UP (M2443), HP_HEPATIC_FIBROSIS (M35270), HP_PERIPORTAL_FIBROSIS (M41235), HP_PORTAL_FIBROSIS (M36886), REACTOME_ACTIVATED_TLR4_SIGNALLING (M15343), GOMF_RAGE_RECEPTOR_BINDING (M18595).

Data were represented as LOG2 fold in a heatmap generated with the Morpheus software (https://software.broadinstitute.org/morpheus/). The mass spectrometry proteomics data have been deposited to the ProteomeXchange Consortium via the PRIDE **(Perez-Riverol Y et *al.* Nucleic Acids Res. 2022)** partner repository with the dataset identifier PXD044031).

➢ Enzyme-linked immunosorbent assay (ELISA)

Cell culture media were collected from wells containing myofibroblasts treated or not treated with GDF 15 (at the concentration of 10 ng/mL). The media underwent centrifugation to remove particulates and assay immediatly. The supernatant was taken to be detected by a

PDGFα/β ELISA kit (Human PDGF-AB, Quantikine ELISA Kit [DHD00C]) by strictly following relevant instructions of kits.

Supplementary figures

- **Fig. S1:**

Comparison of proteomic profiles in perilobular areas from AH vs Cirrh livers

Spatial proteomic analysis shows hierarchical clustering of the most variable proteins between AH and Cirrh extracted from liver perilobular areas

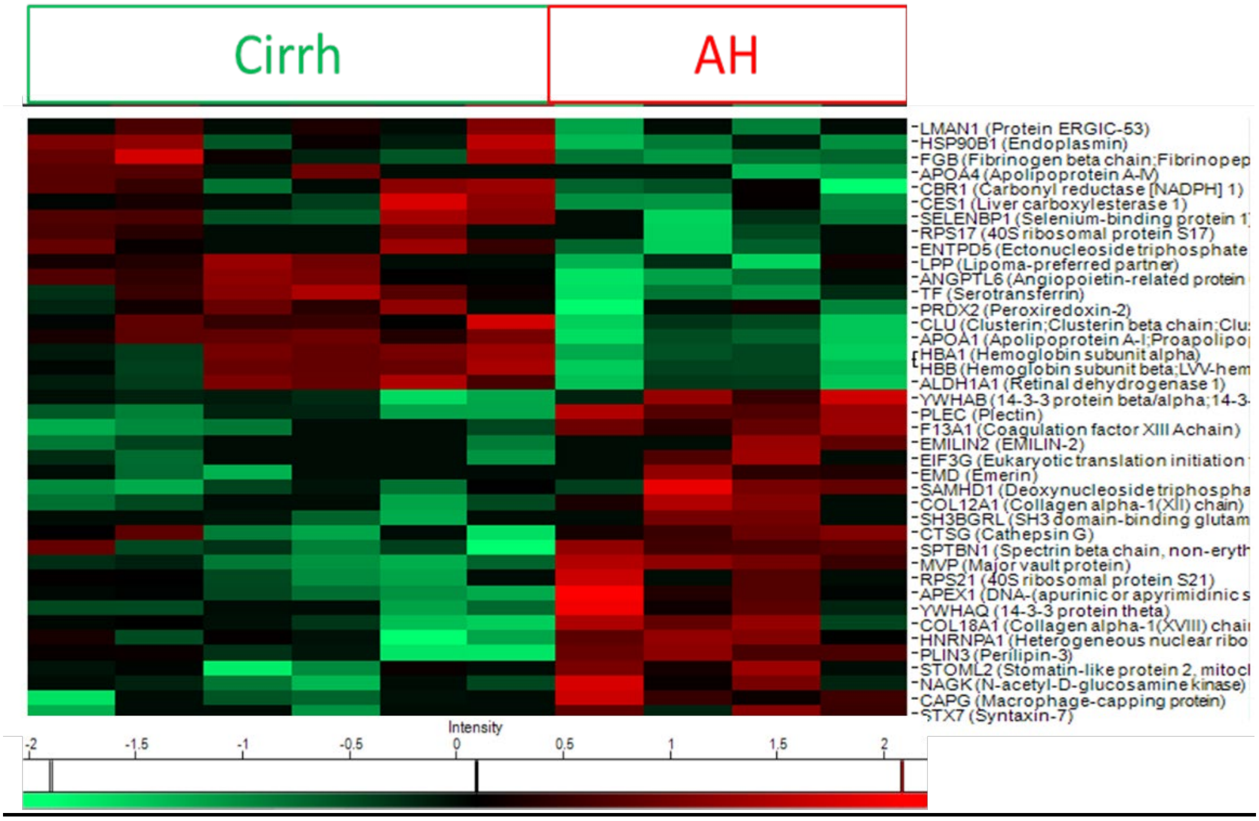

➢ **<u>Fig. S2</u>**

Assessment of CYP3A4 enzymatic activity in organoids obtained from AH or Cirrh livers using the P450-Glo™ assay

The evaluation of CYP3A4 activity in organoids from AH and Cirrh organoids confirms the presence of mature hepatocytes. As expected, CYP3A4 activity was lower in AH organoids than in Cirrh organoids. Statistical significances are indicated (Mann-Whitney U test). * means $p<0.05$; ** means $p<0.01$; *** means $p<0.001$

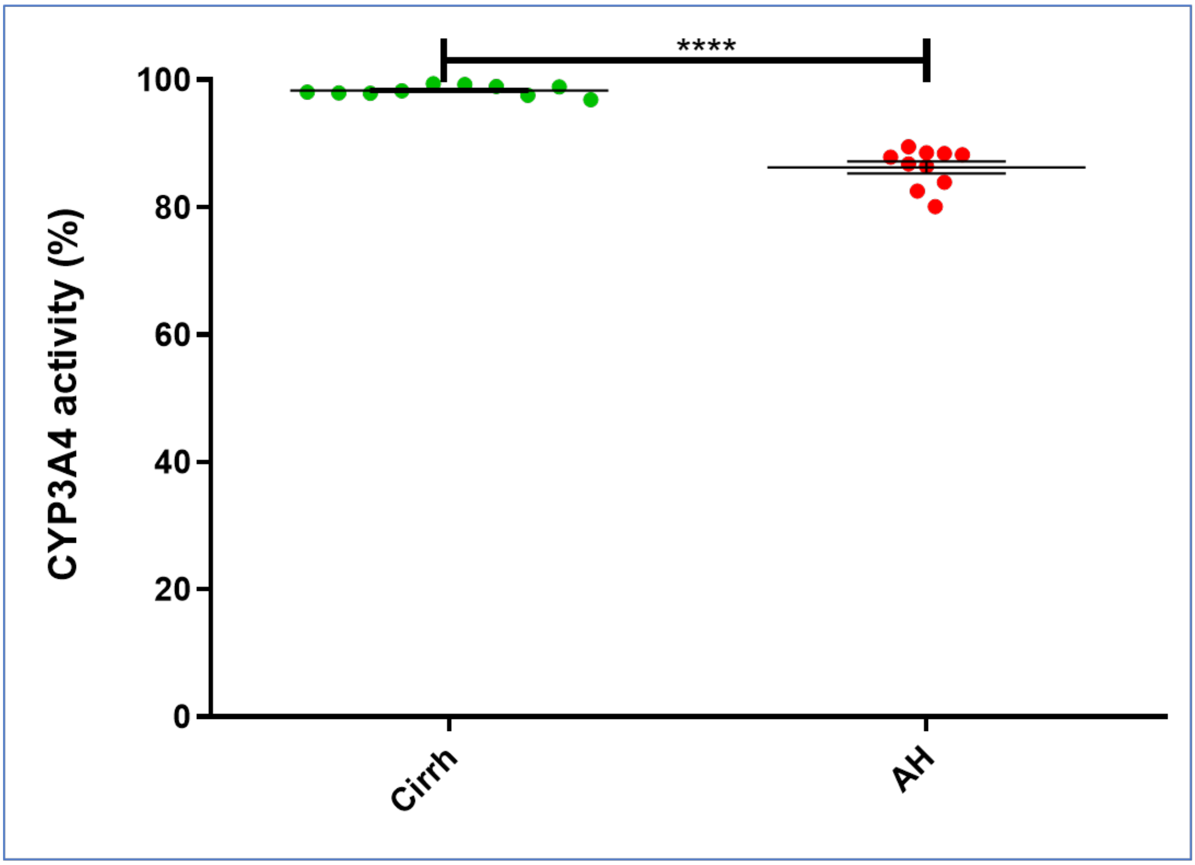

➢ <u>Fig. S3</u>

Expression of YAP and its target genes in transduced and non-transduced Cirrh organoids

Real-time PCRs performed on transduced (with an active YAP, to mimic AH organoids) or non-transduced Cirrh organoids.

mRNA expression levels of the Tag YAPS127A, YAP and its target genes ANKRD1, NUAK2, CYR61 were compared with TBP housekeeping gene (coding for TATA-binding protein)

Statistical significances are indicated (Mann-Whitney U test). * means $p<0.05$; ** means $p<0.01$; *** means $p<0.001$

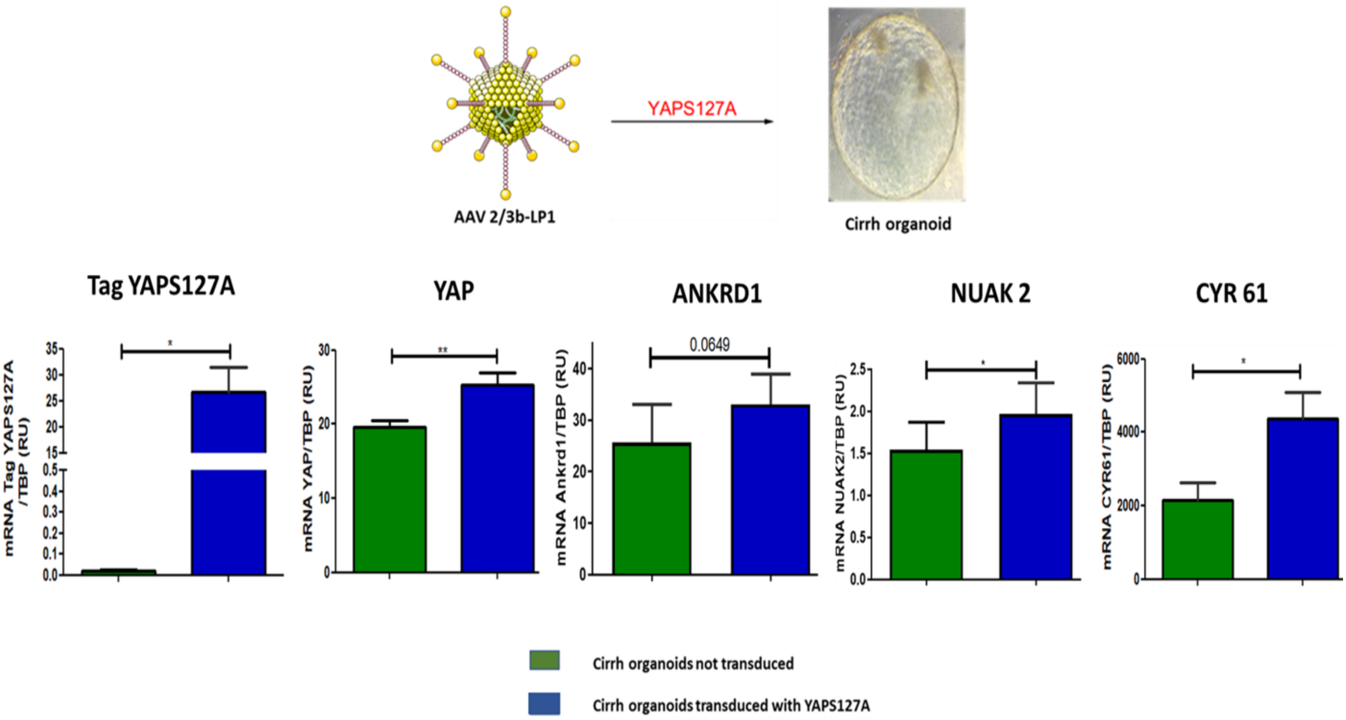

- **<u>Fig. S4</u>**

YAP modulation in human primary hepatocytes

Real-time PCRs performed on transduced (with an active YAP to mimic the aberrant activation of YAP in AH hepatocytes) or non-transduced human primary hepatocytes

mRNA expression levels of YAP, its target genes (ANKRD1, TGFβ2, CYR61, NUAK2 and CTGF), hepatocyte (Albumin, CYP3A4, TAT, Aldolase B) and cholangiocyte (HNF1β, CK 19) markers were compared with TBP housekeeping gene (coding for TATA-binding protein)

Statistical significances are indicated (Mann-Whitney U test). * means $p<0.05$; ** means $p<0.01$; *** means $p<0.001$

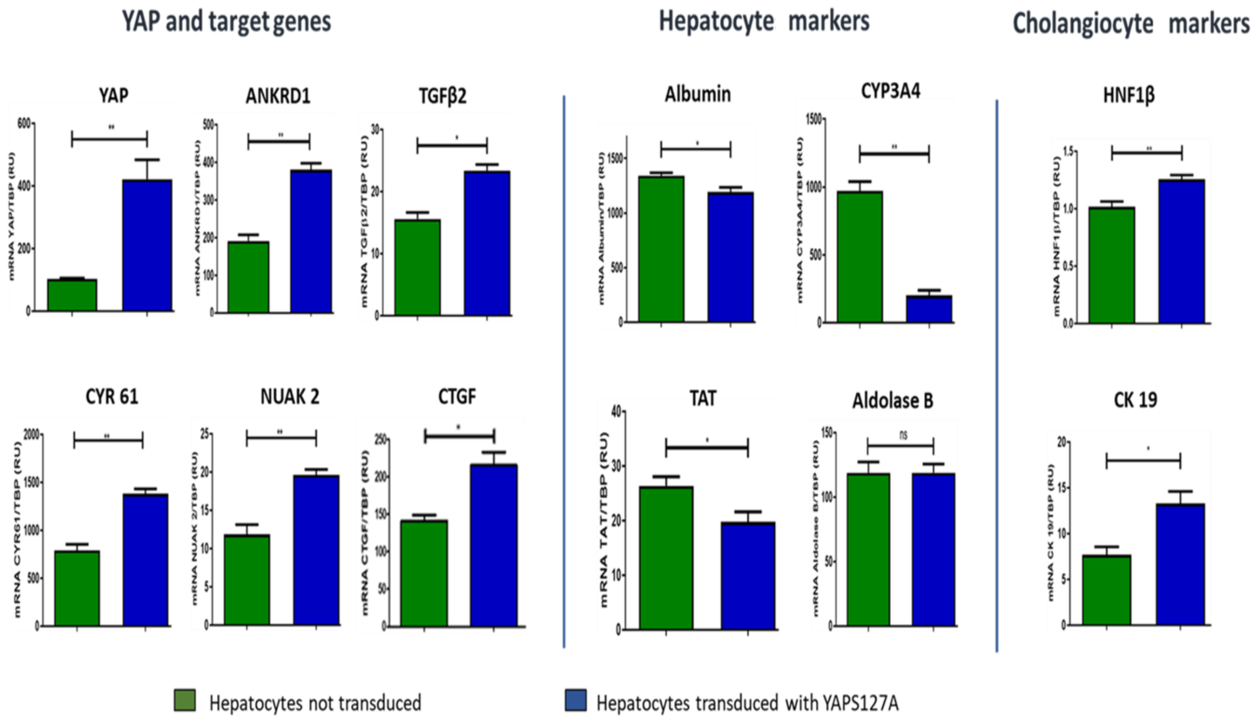

Supplementary tables

- **Table S1**

Primer sequences used for real time RT-PCR

Genes	Forward	Reverse
TBP	TGA-GAA-GAT-GGA-TGT-TGA-GTT-G	AGA-TAG-CAG-CAC-GGT-ATG-AG
αSMA(ACTA2)	CTA-TGC-CTC-TGG-ACG-CAC-AAC-T-	CAG-ATC-CAG-ACG-CAT-GAT-GGC-A
COL1A1	GCC-AAG-ACG-AAG-ACA-TCC-CA	CGT-CAT-CGC-ACA-ACA-CCT-T
PDGFRα	GAT-CTT-TTC-CCT-TGG-TGG-CA	CGT-AGA-CTT-CAC-TGG-TAG-CG
LAMA2	GGC-AAT-CTG-AAT-ACA-CTC-GTG-AC	TGT-GTT-GGT-CCT-CTC-AGC-ATC-C
TIMP1	TGG-AAA-ACT-GCA-GGA-TGG-AC	ATA-AAC-AGG-GAA-ACA-CTG-TGC
MMP9	TTC-TGC-CCG-GAC-CAA-GGA-TA	CCG-GCA-CTG-AGG-AAT-GAT-CT
PDGFα	AGC- GAC-TCC-TGG-AGA-TAG-AC	GAC-AGC-TTC-CTC-GAT-GCT-T
MCP1	GCC-TCC-AGC-ATG-AAA-GTC-TC	AGG-TGA-CTG-GGG-CAT-TGA-T
CCL5	CCTGCTGCTTTGCCTACATTGC	ACACACTTGGCGGTTCTTTCGG
YAP1	TCA-TGC-TTA-GTC-CAC-TGT-CTG-T	TAG-CCC-TGC-GTA-GCC-AGT-TA
Tag YAPS127A	AAGCAAGGCTCGAATCGGTA	TCATGACGGCGTTGAAGAGC
AMOTL2	GCA-AGG-GCT-CTC-TTC-TAG-TG	TGG-GTG-CTC-TGT-CTG-TAG-TC
NUAK2	GAT-GCA-CAT-ACG-GAG-GGA-GATT	ATC-ACG-ATC-TTG-CTG-CTG-TTC-T
TGFβ2	CAC-GAA-CCC-AAA-GGG-TAC-AA	ATA-TAA-GCT-CAG-GAC-CCT-GCT
CTGF	TGG-AGG-AAA-ACA-TTA-AGA-AGG-G	AAG-CTC-AAA-CTT-GAC-AGG-CT
CYR61	TTG-GTA-ACT-CGT-GTG-GAG-ATG	GAA-GAG-GCT-TCC-TGT-CTT-TGG
CYCLIN D1	TGC-ATG-TTC-GTG-GCC-TCT-AAG	TCG-GTG-TAG-ATG-CAC-AGC-TTC-T

- **Table S2**

Antibodies used in immunostaining

Name	Host	Supplier	Reference
**αSMA**	Mouse	DAKO	M0851
**COL1A1**	Mouse	Sigma-Aldrich	SAB1402151
**PDGFRα**	Rabbit	Cell signaling	5241
**YAP**	Rabbit	Cell signaling	≠ 14074
**Albumin**	Goat	Bethyl laboratory	A80-229A
**GDF 15**	Rabbit	Cell signaling	≠ 79996

Supplementary reference

1- Perez-Riverol Y, Bai J, Bandla C, et al. The PRIDE database resources in 2022: a hub for mass spectrometry-based proteomics evidences. *Nucleic Acids Res*. 2022;50(D1):D543-D552. doi:10.1093/nar/gkab1038
